# Evaluation of respiratory disease hospitalisation forecasts using synthetic outbreak data

**DOI:** 10.1038/s43856-026-01802-4

**Published:** 2026-07-29

**Authors:** Grégoire Béchade, Torbjörn Lundh, Philip Gerlee

**Affiliations:** 1 Ecolé Polytechnique, Palaiseau, France; 2 Department of Mathematical Sciences, Chalmers University of Technology & University of Gothenburg, Gothenburg, Sweden

**Keywords:** Epidemiology, Infectious diseases

## Abstract

**Background:**

Forecasts of hospitalisations of infectious diseases play an important role for allocating healthcare resources during epidemics and pandemics. Large-scale analysis of model forecasts during the COVID-19 pandemic has shown that the model rank distribution with respect to accuracy is heterogeneous and that ensemble forecasts have the highest average accuracy.

**Methods:**

Building on that work we generated a maximally diverse synthetic dataset of 324 different hospitalisation time-series that correspond to different disease characteristics and public health responses. We evaluated forecasts from 14 component models and 6 different ensembles.

**Results:**

Our results show that component model accuracy was heterogeneous and varied depending on the current rate of disease transmission. Going from 7 day to 14 day forecasts mechanistic models improved in relative accuracy compared to statistical models. A novel adaptive ensemble method outperforms all other ensembles on synthetic data, and is closely followed by a median ensemble. When evaluated on data from the COVID-19 pandemic, component models performed worse, but the ensemble accuracy was still high, with the median ensemble performing best. We also investigated the relationship between ensemble error and variability of component forecasts and show that the coefficient of variation is predictive of future error.

**Conclusions:**

Our findings have the potential to improve epidemic forecasting, in particular the adaptive ensemble and the ability to assign confidence to ensemble forecasts at the time of prediction based on component forecast variability.

## Plain Language Summary

Forecasts of hospital admissions during epidemics help healthcare systems prepare for increased demand. However, it is often unclear which forecasting methods work best under different outbreak conditions. In this study, we created artificial, yet realistic, data on the number of hospitalsed patients of hypothetical respiratory diseases using a computer model based on COVID-19 transmission. We then tested 14 different forecasting models and several methods for aggregating forecasts into so called ensemble forecast, e.g. by taking the mean of the forecasts from individual models. We found that no single model consistently performed best. Instead, combining forecasts from several models usually gave more reliable predictions. We also developed a new type of ensemble method that changes how much it trusts different models depending on how fast the disease is spreading. This method performed especially well on simulated outbreaks and showed promising results on real COVID-19 data. These findings could help improve epidemic forecasting and support better planning of healthcare resources during future outbreaks.

## Abstract (web summary)

Béchade et al., generate and evaluate forecasts from 14 component models and 6 ensemble approaches using 324 synthetic hospitalisation time series representing diverse epidemic and public health scenarios. Forecast accuracy varies with transmission dynamics and forecast horizon, while ensemble methods consistently perform well, with an adaptive ensemble and median ensemble achieving the highest accuracy and forecast uncertainty helping predict future error.

## Introduction

Prediction of future outcomes, such as incidence, hospital admissions and mortality, plays an important role in infectious disease epidemiology^1^, both in terms of short-term prediction or forecasts and longer-term scenario projections^2^. The time-scales relevant for forecasting and projections depends on the characteristics of the disease, but it is generally acknowledged that the accuracy of forecasts degrade rapidly beyond a month^3^. This depends both on the inherently non-linear nature of disease transmission and on potential rapid changes in processes relevant for transmission, e.g. altered contact rates due to enforced or voluntary social distancing^4,5^.

Forecasts of key epidemiological variables have played an important role in a number disease outbreaks including Zika virus^6^, Ebola^7^, Seasonal Influenza^8^ and COVID-19^3^. The utility of infectious disease forecasts ranges from informing decision-makers about the future developments of the epidemic to the distribution of medical supplies and supporting the allocation of healthcare resources.

Forecasting models require historical data in order to produce useful forecasts. While mortality data typically remains stable during an epidemic it is often subject to reporting delays and in addition there is typically a delay of several weeks from infection to death, which can hamper forecasting efforts. Incidence data on the other hand does not suffer from this drawback, but is instead affected by testing strategies, which can vary significantly during the course of an epidemic. It has been argued that hospital admission present a useful middle-ground since admission criteria are more stable and forecasts of hospitalisations are valuable for regional decision-makers and allocation of healthcare resources, such as ICU-beds^4^.

For these purposes a whole range of different forecasting models have been utilised, ranging from statistical models, mechanistic/compartmental models in terms of ordinary differential equations to agent-based models^9^. This diversity of approaches became obvious during the COVID-19 pandemic and was particularly highlighted through the efforts of a number of forecast hubs where research groups and individuals alike could submit forecasts on a regular basis as well as documentation and code of their model^10^. An example of this was the US COVID-19 Forecast Hub^11^ which operated from spring 2020 until the autumn 2023 and focused on forecasts of mortality and hospitalisations at both the national and state level in the US. Another example is the FluSight Forecast Hub which has been operational since the 2013/2014 influenza season^12^.

An evaluation carried out on forecasts of mortality submitted to the US Forecast Hub showed large heterogeneity in performance among the 27 models that were considered^3^. Approximately two-thirds of the models performed better than a naïve baseline model and no single model outperformed the others. Instead, it was observed that an ensemble of model forecasts performed best on average. The hub ensemble was to begin with formed by taking an unweighted average of point predictions and quantile levels of the submitted forecasts, but was later changed into an ensemble formed from the median of the component forecasts.

Other methods for forming an ensemble have also been considered, e.g. optimising the weights of the models with respect to the performance of component models on historical data^13^. While this improves performance relative to an unweighted mean the performance is similar to a median ensemble^14^, which is both conceptually easier to communicate and computationally easier to calculate. However, there is currently no theoretical basis for choosing component models in an optimal way, nor a theoretical understanding of why certain ensembles perform better than others.

Thus, there is a need to better understand the performance of both individual models and ensemble forecasts. One approach to this problem is to evaluate models and ensembles in a large-scale epidemic dataset, and while the evaluation of the US Forecast Hub contains large number of individual forecasts they are not independent since they concern geographical locations with similar transmission dynamics. Our goal here is to develop a novel method for evaluating epidemiological forecasting models for emergent outbreaks, that utilises synthetically generated diverse data that reflects both different disease characteristics and responses to the epidemic in terms of time-dependent mobility.

In order to further our understanding of epidemic forecasting we present a large-scale and maximally diverse dataset of hospital admissions against which forecast models can be tested. We have characterised the performance of 14 different component models ranging from statistical to mechanistic models and covering both univariate and multivariate models. We also evaluated six different ensemble models and present a novel adaptive ensemble which utilises information about the current transmission dynamics. Lastly, our large-scale approach offers new insight into the relationship between the error of ensemble forecasts and the variance of component forecasts, information that can be used to judge the reliability of ensemble forecasts.

## Methods

In this section, we provide details of the data generation, the forecast models, performance metrics and ensemble forecasts. All code and data are available at: Zenodo^15^.

### Data generation—Covasim

To generate the synthetic outbreak data Covasim^16^, an agent-based model was used that can simulate the transmission of a respiratory infectious disease in a population. This model takes as an input disease- and population-specific parameters such as the population size, the age distribution, the transmission probability and age-dependent severity, and outputs a complete description of the outbreak, such as time-series of the number of severe and asymptomatic cases, and also the effective reproduction number.

**Fig. 1:**
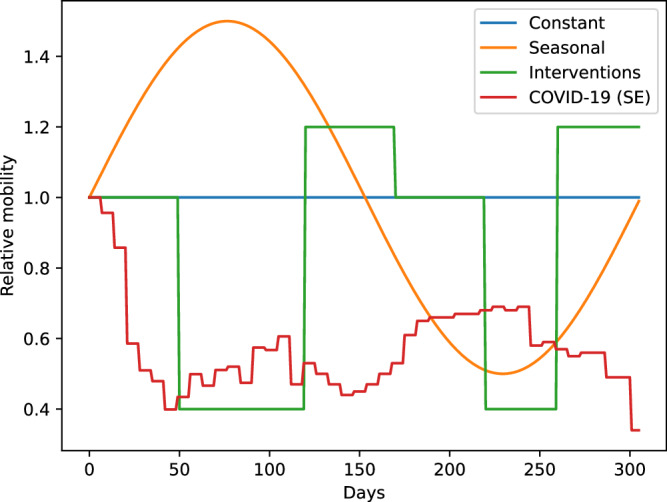
Time-dependent mobility. The different time-dependent mobilities that were used as input to Covasim.

Covasim allows for the generation of diverse outbreaks, thanks to the large number of parameters that can be given as input to the model, but also due to the possibility to introduce interventions that can be planned by the user. For this work, we make use of non-pharmaceutical interventions in terms of a time-dependent mobility which modulates the transmission probability of all contacts. We consider four different mobilities: (i) actual mobility data from Västra Götaland Region in Sweden during the COVID-19 pandemic^5^, (ii) seasonal mobility modelled as a sine curve, (iii) rapid changes to the mobility meant to represent mandatory lock-downs and (iv) a constant mobility (see Fig. 1).

To begin with we used default settings for all parameters meaning that the disease being modelled is similar to COVID-19. The location was set to ‘Sweden’ which implies that the age distribution, contact networks and other population properties are set to match Swedish data. To reduce computational time we set the population size to 10^6^ individuals.

We assume that all severe and critical cases require hospital care and consider the daily number of critical and severe cases as the number of hospitalised each day. This time-series will be target for the forecast models.

### Parameter sensitivity analysis

In order to generate a diverse set of outbreaks we need to define a metric that quantifies the difference between two outbreaks (in terms of time-series of hospitalised per day). Initial testing showed that it was not sufficient to simply take the absolute or squared distance between the time-series representing the hospitalisations of the two outbreaks. Given two time-series of hospitalisations *Y*_1_ and *Y*_2_ we opted for the following metric:

1 $${\hskip -12pt} \begin{array}{rc}&{{\mathcal{L}}}({Y}_{1},{Y}_{2})=\parallel \left(\parallel {Y}_{1}-{Y}_{2}{\parallel }_{{L}_{1}},\parallel {Y}_{1}^{{\prime} }-{Y}_{2}^{{\prime} }{\parallel }_{{L}_{1}},\parallel {Y}_{1}^{{\prime\prime} }-{Y}_{2}^{{\prime\prime} }{\parallel }_{{L}_{1}},\frac{\max ({Y}_{1})}{\max ({Y}_{2})},\frac{\max (Y^{\prime}_{1}) }{\max (Y^{\prime}_{2})},\frac{\max (Y^{\prime\prime}_{1})}{\max (Y^{\prime\prime}_{2})}\right){\parallel }_{{L}_{2}},\end{array}$$

where ${Y}_{i}^{{\prime} }$ and ${Y}_{i}^{{\prime\prime} }$ are the first and second numerical derivatives and $\parallel Y{\parallel }_{{L}_{1}}={\sum}_{i}| {Y}^{i}|$ is the sum of absolute values of the vector *Y* and $\parallel X{\parallel }_{{L}_{2}}={\sum}_{i}{X}_{i}^{2}$. It can be noted that ${{\mathcal{L}}}$ is similar to the Sobolev norm $\parallel {Y}_{1}-{Y}_{2}{\parallel }_{{W}^{2,1}}$ with the additional terms accounting for maximal difference in amplitude.

As Covasim has a large set of inputs parameters, a first subset of key parameters was identified: the *spread* parameters and the *severity* parameters, which are parameters related to disease transmission and severity. The *severity* parameters are the 4 parameters that correspond to the probability for an individual to move from one disease compartment to another. The *spread* parameters are 9 parameters that represent the distribution of probability of the time spend by an agent in a compartment (such as infected, crictical…) once it has been entered. The value of each individual is drawn from a log-normal distribution, and the *spread* parameters correspond to the mean of this log-normal distribution. All the parameters have a default value of 1, which correspond to keeping the reference value.

This set of 13 parameters is denoted *S*. In order to test the forecasting models on a large, but still manageable set of different pandemics we decided to vary four parameters and that these should each take three different values in [0.5, 1, 2], leading to a set of 81 pandemics. To select the 4 parameters in *S* that generated the most diversity with respect to ${{\mathcal{L}}}$ out of the 13 possible is equivalent to solving the following problem:

2 $${s}_{{{\rm{opt}}}}={{argmax}}_{{{s}} \subset {{S}},| {{s}}| =4}\,{{\mathcal{H}}}({{s}}),$$

with ${{\mathcal{H}}}(s)={\sum}_{{p}_{1},{p}_{2}\in {{{\mathcal{P}}}}_{g}(s)}{{{\mathcal{L}}}}_{2}({p}_{1},{p}_{2})$, and ${{{\mathcal{P}}}}_{g}(s)$ the set of the 81 pandemics generated with the 4 parameters of *s*. However, generating an outbreak with Covasim is time-consuming, and it is not possible to compute the diversity of each set of 4 parameters *s* included in *S* (the set of all 13 parameters) in reasonable time.

An MCMC algorithm^17^ was therefore implemented, to perform a clever grid search on the different subsets *s* ⊂ *S* of parameters. After 200 iterations of the MCMC algorithm, the set of parameters that maximised the diversity was found to be [sym2sev, asym2rec, rel_symp_prob, rel_severe_prob].

They correspond respectively to the mean of the log-normal distribution representing the time spent in the compartment ‘symptomatic’ before moving into the compartment ‘severe’, the mean of the log-normal distribution representing the time spent in the compartment ‘asymptomatic’ before moving into the compartment ‘recovered’, to the scale factor for proportion of symptomatic cases and to the scale factor for proportion of symptomatic cases that become severe. (see^16^). These ranges are biologically plausible across acute respiratory infections, where progression times and severity proportions vary by at least a factor of two depending on pathogen and host.

Each of the 4 parameters was set to three different values: [0.5, 1, 2] and we considered the four time-dependent mobilities described above resulting in 81 × 4 = 324 different outbreaks. All outbreaks were simulated until everyone in the population had recovered. This lasted less than 300 days for a majority outbreaks and to make the evaluation of forecasts comparable across outbreaks all hospitalisation curves were terminated at 300 days. The resulting outbreaks can be seen in Supplementary Fig. 9.

### Forecast models

In this study, we define a forecast model as a function *h*_*θ*_(*i*), which equals the number of hospitalised cases at day *i* since the outbreak began, with parameters *θ* that are estimated by training on the data ${{\mathcal{D}}}$. In the training phase, $\hat{\theta }$, an estimator of *θ* is computed from ${{\mathcal{D}}}$, and used for the prediction.

We considered two types of models: univariate models which are only trained on the time series we want it to predict (the number of hospitalized in our case), and multivariate models that are trained on the time series we want to predict, but also on other time series that can be relevant to predict the number of hospitalized: the mobility and incidence (new cases/day).

Another way to classify the models is according to their underlying structure. We will consider statistical models that use time as a covariate, autoregressive models that use data from previous days as covariates and mechanistic models that are formulated as systems of coupled ordinary differential equations.

During the training or prediction phase computations sometimes fail (e.g. due to non-invertible matrices). Instead of outputting a missing value, we let the model output the value of the Moving average-model (see below), which can be interpreted as a naive fallback when computation fails.

For a forecast that is made on day *i* that reaches *r* days into the future the model is trained on the set ${{{\mathcal{D}}}}_{i}={\{{Y}_{j}\}}_{j = 0}^{i}$. The point forecast is given by ${\hat{Y}}_{r}={h}_{\hat{\theta }}(r)$ where $\hat{\theta }$ are the estimated parameter values of the model. To each point forecast we associate (1 − *α*) prediction intervals, where *α* = [0.02, 0.05, 0.1, 0.2, 0.3, 0.4, 0.5, 0.6, 0.7, 0.8, 0.9], making the forecasts probabilistic.

#### Statistical models

**Exponential regression:** This models assumes that the number of hospitalised cases follows *h*(*t*, *a*, *b*, *c*) = *ae*^*b*(*t*−*c*)^, where *θ* = (*a*, *b*, *c*) are the parameters of the model and *t* is the number of days since the start of the outbreak. The parameters are estimated using least squares optimisation with the SciPy-function curve_fit and prediction intervals are computed using the Delta method^18^.

**Multivariate exponential regression:** This model is similar to the univariate case and assumes that $h(t,a,b,c,d,e)=a{e}^{(b+d{m}_{t}+e{i}_{t})(t-c)}$ where *d* and *e* are additional parameters, *m*_*t*_ is the mobility at day *t* and *i*_*t*_ is the incidence at day *t*. Again, we make use of least squares optimisation and the Delta method. When predictions are made we assume that the mobility and incidence take last known value for all future dates.

#### Autoregressive models

**Moving average:** This is the simplest of all models and serves as our baseline-model. The prediction is constant and equals the mean number of hospitalised cases in the past seven days. The prediction interval of the prediction is calculated as the standard deviation during the past seven days.

**ARIMA:** The ARIMA(*p*, *d*, *q*) model is the sum of an AR(*p*) and a MA(*q*) model applied on the time series differentiated *d* times. It follows the equation:

3 $${Y}_{t}^{d}=\alpha +{\sum }_{i=1}^{p}{\beta }_{t-i}\,{Y}_{t-i}^{d}\,+\,{\sum }_{j=1}^{q}{\phi }_{t-j}\,{\epsilon }_{t-j},$$

where ${Y}_{t}^{d}$ is the time series at time *t*, *d* is the order of the differentiation, *α* is a constant, *p* is the order of the autoregressive part, *q* is the order of the moving average part and *ϵ*_*t*−*j*_ is the difference between the prediction of the model and the real value at time *t* − *j*. The coefficient are estimated through maximum likelihood estimation. This method is implemented in the statsmodels library, which directly provides prediction intervals. We performed a grid search on a single pandemic to identify the combination of parameters that would optimize the prediction accuracy. We found an optimal value for *p* = 3, *d* = 0, *q* = 3.

**VAR:** The VAR model is a multivariate AR-model, where several variables are predicted. This model exploits the correlation between variables. Let *Y*_1,*t*_, …, *Y*_*k*,*t*_ be the times series (in our case, *k* = 3 and they correspond to the number of hospitalized, the number of infected and the mobility at day *t*).

4 $$\begin{array}{l}{{\rm{VAR}}}(p):\left(\begin{array}{l}{Y}_{1,t}\\ {Y}_{2,t}\\ \vdots \\ {Y}_{k,t}\end{array}\right)=\left(\begin{array}{l}{c}_{1}\\ {c}_{2}\\ \vdots \\ {c}_{k}\end{array}\right)+\left(\begin{array}{llll}{\phi }_{11,1}&{\phi }_{12,1}&\cdots \,&{\phi }_{1k,1}\\ {\phi }_{21,1}&{\phi }_{22,1}&\cdots \,&{\phi }_{2k,1}\\ \vdots &\vdots &\ddots &\vdots \\ {\phi }_{k1,1}&{\phi }_{k2,1}&\cdots \,&{\phi }_{kk,1}\end{array}\right)\left(\begin{array}{l}{Y}_{1,t-1}\\ {Y}_{2,t-1}\\ \vdots \\ {Y}_{k,t-1}\end{array}\right)\\ +\cdots +\left(\begin{array}{llll}{\phi }_{11,p}&{\phi }_{12,p}&\cdots \,&{\phi }_{1k,p}\\ {\phi }_{21,p}&{\phi }_{22,p}&\cdots \,&{\phi }_{2k,p}\\ \vdots &\vdots &\ddots &\vdots \\ {\phi }_{k1,p}&{\phi }_{k2,p}&\cdots \,&{\phi }_{kk,p}\end{array}\right)\left(\begin{array}{l}{Y}_{1,t-p}\\ {Y}_{2,t-p}\\ \vdots \\ {Y}_{k,t-p}\end{array}\right)+\left(\begin{array}{l}{\epsilon }_{1,t}\\ {\epsilon }_{2,t}\\ \vdots \\ {\epsilon }_{k,t}\end{array}\right)\end{array}$$

Again, the *ϕ*_*i*,*j*,*k*_ and *c*_*i*_ are estimated through maximum likelihood estimation with the statsmodel library. In order to produce a forecast at day *t*, *n* days ahead the VAR-model predicts all three variables (hospitalisations, incidence and mobility) one day ahead and uses these predicted variables to predict two days ahead. This process is repeated *n* times until *Y*_1,*t*+*n*_, *Y*_2,*t*+*n*_, *Y*_3,*t*+*n*_ have been computed. The prediction intervals are also directly provided by the library.

**Linear regression:** This is an AR-model where the parameters are estimated using linear regression. In order to implement this model we converted the time-series *Y*_*t*,*t*∈{1, …*n*}_ into a training set (*X*_*i*_, *Y*_*i*_) such that:  ∀ *i* ∈ {1, . . . , *n*}, *X*_*i*_ = (*Y*_*i*−1_, *Y*_*i*−2_, . . . , *Y*_*i*−20_), which implies that the order of the model equals *p* = 20. The model was implemented using the scikit-learn package.

The confidence interval for the linear regression prediction was computed as follows: Let us suppose that the data follows a linear regression model: *Y* = *Xβ* + *ϵ*, with $Y\in {{\mathbb{R}}}^{n}$, $X\in {{\mathbb{R}}}^{n\times d}$, $\beta \in {{\mathbb{R}}}^{d}$ and $\epsilon \sim {{\mathcal{N}}}(0,{\sigma }^{2})$. The least square estimator of *β* is $\hat{\beta }={({X}^{T}X)}^{-1}{X}^{T}Y$. If we create a new matrix $\tilde{X}$ with unseen data, we can perform the prediction as follow:

5 $$\begin{array}{ll}\tilde{Y}=\tilde{X}\hat{\beta }\\ =\tilde{X}{({X}^{T}X)}^{-1}{X}^{T}Y\\ =\tilde{X}{({X}^{T}X)}^{-1}{X}^{T}(X\beta +\epsilon )\\ =\tilde{X}\beta +\tilde{X}{({X}^{T}X)}^{-1}{X}^{T}\epsilon .\end{array}$$

This implies that $\tilde{Y}$ follows a normal distribution of expected value $\tilde{X}\beta$ and variance $\tilde{X}{({X}^{T}X)}^{-1}{\tilde{X}}^{T}{\sigma }^{2}$.

**Bayesian regression:** This model is similar to linear regression, and also implemented using the scikit-learn package, but the parameters are estimated using Bayesian ridge regression.

The Bayesian ridge regression model treats the problem of predicting the future number of severe cases as a linear regression problem, where past observations serve as covariates. The default setting is to use data stretching 20 days into the past to predict the number of severe cases tomorrow, i.e.

6 $${\hat{Y}}_{t+1}={w}_{0}+{w}_{1}{Y}_{t}+\cdots +{w}_{19}{Y}_{t-19}+{\varepsilon }_{t},$$

where ${\hat{Y}}_{t+1}$ is the predicted number of severe cases at day *t*, *Y*_*t*−*i*_ is the number of severe cases *i* days prior, *w*_*i*_ are the weights, which are estimated using historical data, and *ε*_*t*_ is Gaussian noise with mean zero and variance *λ*^−1^.

The weights follow a Gaussian prior:

7 $$w \sim {{\mathcal{N}}}(0,{\alpha }^{-1}I),$$

where *α* is the prior precision, controlling the regularization strength.

The likelihood of the observed data given the weights is Gaussian:

8 $$y| X,w,\lambda \sim {{\mathcal{N}}}(Xw,{\lambda }^{-1}I),$$

where *X* refers to the historical data.

Both the weights and the values of *α* and *λ* are estimated from the data using empirical Bayes (maximizing the marginal likelihood). The posterior distribution of the weights is also Gaussian, combining information from the prior and the likelihood.

#### Mechanistic models

**SIRH:** The SIRH-model is an extension of the classic compartmental SIR (Susceptible-Infectious-Recovered) model used to describe the spread of infectious diseases. In the SIRH model, a fourth compartment, *H* for Hospitalised, is added. The evolution of the number of individuals in each compartment is described by a system of coupled ordinary differential equations:

9 $$\left\{\begin{array}{rlr}&\frac{dS}{dt}=-\beta \frac{SI}{N}&\\ &\frac{dI}{dt}=\beta \frac{SI}{N}-{\gamma }_{i}I-hI\\ &\frac{dR}{dt}={\gamma }_{i}I+{\gamma }_{h}H\\ &\frac{dH}{dt}=hI-{\gamma }_{h}H\end{array}\right.$$

At *t* = 0, the values of (*S*_0_, *I*_0_, *R*_0_, *H*_0_) are fixed to (10^6^ − 1, 1, 0, 0, ), corresponding to a single infectious individual in a otherwise susceptible population. The equations were solved using the SciPy-function odeint.

To train this model, we minimize the least squares error between the number of hospitalised cases in the training data and *H*(*t*) with respect to *θ* = (*β*, *γ*_*i*_, *γ*_*h*_, *h*), using curve_fit. We implemented four version of the SIRH-model in which either *γ*_*i*_, *γ*_*h*_ were fixed or subject to optimisation. (see the Table 1). If fixed they were set to *γ*_*i*_ = 1/8 and *γ*_*h*_ = 1/18. These values agree with the mean number of days for recovery for regular (8 days) and severe cases (18 days) in Covasim, and from the point of view of the forecaster corresponds to partial knowledge about the disease. The information is partial since the infectiousness *β* and rate of hospitalisation *h* are always unknown to the forecaster.

**Table 1:** Difference between the SIRH models with respect to the *γ*-parameters

	*γ*_*i*_	*γ*_*h*_
SIRH1	1/8	1/18
SIRH2	1/8	free
SIRH3	free	1/18
SIRH4	free	free

For a prediction made at day *t* with reach *r* we solve the model numerically with the above mentioned initial condition with the parameters $\hat{\theta }$ computed during the training phase. The prediction is shifted so that it equals the observed number of hospitalised cases at day *t*, i.e. *H*(*t*) = *Y*_*t*_. The prediction interval of the prediction is computed using the Delta method and numerical differentiation.

**SIRH-Multi:** We also implemented two SIRH-models where the mobility data influences the contact rate. We assume that *β* varies with the time as a linear combination of the mobility: *β*_*t*_ = *a* + *b* × *m*_*t*_, similar to what was used in ref. ^5^. When solving the model beyond the day of forecast we make use of a linear extrapolation of the mobility since its future values are unknown to the forecaster.

We consider two versions of the SIRH-Multi model in which the values of *γ*_*h*_ and *γ*_*i*_ are either fixed or subject to optimisation. SIRH-Multi1 refers to the model in which *γ*_*h*_ and *γ*_*i*_ are free and SIRH-Multi2 refers to the model in which they are fixed to *γ*_*i*_ = 1/8 and *γ*_*h*_ = 1/18.

**SEIR-Mob:** This model is adapted directly from^5^ and consists of an SEIR-model with time-dependent mobility of the form *β*_*t*_ = *a* + *b* × *m*_*t*_. The model assumes that a fraction *p* of the infected cases become hospitalised with a delay of 21 days. To train the model, we minimize the least squares error between the number of hospitalised cases in the training data and the model solution with respect to *θ* = (*a*, *b*, *p*), using curve_fit. Model prediction intervals are computed as for the SIRH-models. As above, we make use of a linear extrapolation of the mobility since its future values are unknown to the forecaster.

### Performance metrics

Three metrics were used to assess the performance of the models. The first metric is the Weighted Interval Score (WIS), which is a metric commonly used in forecast evaluation^3^.

Let *α* be in $\left.\right]0,1\left[\right.$. Let $\hat{y}$ be the prediction of the model and *y* the real value. Let [*l*, *u*] be the (1 − *α*) prediction interval of the prediction. The Interval Score (*IS*) is defined as

10 $$I{S}_{\alpha }([l,u],y)=\frac{2}{\alpha }\times ({{\mathbb{1}}}_{\{y < l\}}(l-y)+{{\mathbb{1}}}_{\{y\ > \ u\}}(y-u)+(u-l)).$$

This metric consists of three terms: a term of overprediction that punishes a model with a prediction interval at level *α* which is above the real value, a term of underprediction that punishes a model whose prediction interval is under the real value, and a term of range, that punishes too wide prediction intervals.

Let ${({\alpha }_{k})}_{k\in \{1,\ldots ,K\}}\in \left.\right]0,1\left[\right.$ ^*K*^ be a sequence of significance levels. The WIS is now defined as

11 $${{\rm{WIS}}}\left([l,u],\hat{y},y\right)={w}_{0}| y-\hat{y}| +{\sum }_{k=1}^{K}{w}_{k}I{S}_{{\alpha }_{k}}\left([l,u],y\right),$$

with weights ${({w}_{k})}_{k\in \{1,\ldots ,K\}}\in {{\mathbb{R}}}_{+}^{K}$ chosen by the user.

According to previous literature^3^, we decided to set ${({\alpha }_{k})}_{k\in \{1,\ldots ,K\}}=[1,0.02,0.05,0.1,0.2,0.3,0.4,0.5,0.6,0.7,0.8,0.9]$ and $\forall k\in \{1,\ldots ,K\},{w}_{k}=\frac{{\alpha }_{k}}{2}$.

The second metric is the Root Mean Square Error (RMSE). With the same notations as above, we define the RMSE as

12 $${{\rm{RMSE}}}(\hat{y},y)=\sqrt{{(y-\hat{y})}^{2}}$$

This metric focuses on the point prediction, and does not take into account the prediction intervals.

The third metric is the Mean Absolute Percentage Error (MAPE), which is calculated by averaging the absolute percentage error

13 $${{\rm{APE}}}(\hat{y},y)=| \frac{y-\hat{y}}{y}| .$$

Given the presence of outlier forecast, which occur when model calibration fails to converge, we consider a trimmed average of MAPE where the top 5% of measurements for each model are discarded.

The models were tested on all the 324 pandemics, on 14 data points different (at days 20, 40, 60, ..., 280). For each individual point, the models were trained on the previous days of the pandemic. A 7 and 14 days ahead point prediction was computed, and [0.02, 0.05, 0.1, 0.2, 0.3, 0.4, 0.5, 0.6, 0.7, 0.8, 0.9] prediction-intervals were constructed. The WIS and the RMSE of these predictions were then calculated. For the analysis of the results, we decided to remove the points for which the number of hospitalized was below 10 (i.e., less than 10^−4^ hospitalized per 100,000 citizens). The reason being that it is of little use to assess the performances of the models during the period in which hospitalisations and incidence are low. Not removing those points would lead to biased results, as points of low hospitalisations represent 44% of the dataset, and we would have concluded that the best model is the one that performs well when there is no or very little transmission.

### Classifying stages of the epidemic

In order to compare the performance of the models at different stages of an outbreak, we classified each day of the outbreak into five categories based on the effective reproduction number *R*_eff_. This number was extracted from the Covasim simulations. The classification was made according Table 2.

**Table 2:** Classification according to reproduction number

Condition	Classification
*R*_eff_ < 0.5	Minimal transmission
0.5 ≤ *R*_eff_ < 0.8	Low transmission
0.8 ≤ *R*_eff_ < 1.2	Stable
1.2 ≤ *R*_eff_ < 3	High transmission
*R*_eff_ ≥ 3	Very high transmission

Among all 2549 points where model predictions were evaluated, 698 were classified as ‘minimal transmission’, 579 as ‘low transmission’, 388 as ‘stable’, 752 as ‘high transmission’, 132 as ‘very high transmission’.

### Perturbing additional data streams

We consider four different perturbations of the mobility and incidence data (shown in Supplementary Fig. 10):

- The temporal resolution of the incidence and mobility is reduced and only updated every *n* days, where *n* ranges from 2 to 31.
- The mobility data *m*_*t*_ is perturbed by adding uncorrelated Gaussian noise, i.e. ${\tilde{m}}_{t}={m}_{t}+X$, where *X* ~ *N*(0, *σ*^2^), where 10^−3^ < *σ*^2^ < 10^−1^.
- The incidence data is perturbed by adding noise from a Poisson-Gamma mixture, i.e. ${\tilde{I}}_{t} \sim {{\rm{Poisson}}}({{\Gamma }})$ and Γ ~ Gamma(*k*, *I*_*t*_/*k*), which has the properties ${\mathbb{E}}[{\tilde{I}}_{t}]={I}_{t}$ and ${{\rm{Var}}}({\tilde{I}}_{t})={I}_{t}+{I}_{t}^{2}/k$. We vary *k* in the range 10 < *k* < 1000.
- The incidence data and mobility is delayed by *n* days so that ${\tilde{m}}_{t}={m}_{t-n}$ and ${\tilde{I}}_{t}={I}_{t-n}$.

### The ensemble model

This section describes how the ensemble forecast were computed. The dataset of hospitalisation curves was randomly split into a training set and evaluation set where each was assigned to the training set with probability 0.5. Training of the ensemble weights was only performed on the training set.

**Average Ensemble (EnsAvg):** this ensemble takes an unweighted mean of all component models to calculate the point prediction. The positions of the upper and lower prediction intervals for each significance level were also computed as an unweighted mean.

**Median Ensemble (EnsMedian):** this ensemble uses the median of all point predictions. The positions of the upper and lower prediction intervals for each significance level were also computed using the median.

**Regression Ensemble (EnsReg):** this ensemble takes a weighted mean of the component point predictions plus an intercept

14 $${Y}_{t}^{E}={w}_{0}+{\sum }_{k=1}^{14}{w}_{k}{\hat{Y}}_{t}^{k}$$

The weights *w*_*k*_ and intercept *w*_0_ were obtained using linear regression where the actual hospitalisation on day *t* is the target variable. The positions of the upper and lower prediction intervals for each significance level were also computed according to (14).

**RMSE-optimised Ensemble (EnsRMSE):** This ensemble is similar to EnsReg, but considers weights that sum to unity and no intercept. The weights are obtained by minimising the function

15 $${E}_{{{\rm{RMSE}}}}({{\bf{w}}})={\sum }_{i=1}^{{N}_{T}}{\sum }_{j=1}^{15}{{\rm{RMSE}}}\left({Y}_{i,j},{\sum }_{k=1}^{14}{w}_{k}{\hat{Y}}_{i,j}^{k}\right)$$

subject to the constraint ${\sum}_{k}{w}_{k}=1$. Here the double sum runs over all outbreaks in the training set and across all forecasts within a given outbreak, ${\hat{Y}}_{i,j}$ is the observed number of hospitalised cases and ${\hat{Y}}_{i,j}^{k}$ is the forecast of model *k*. The opimisation problem is solved using the SciPy-function minimize with the trustconst-method and an initial guess equal to 1/14 for all *w*_*k*_.

**WIS-optimised Ensemble (EnsWIS):** This ensemble is similar to EnsRMSE with the difference that it is the WIS which is optimised. Therefore we minimise the function

16 $${E}_{{{\rm{WIS}}}}({{\bf{w}}})={\sum }_{i=1}^{{N}_{T}}{\sum }_{j=1}^{15}{{\rm{WIS}}}\left(\left[{\sum }_{k=1}^{14}{w}_{k}{l}_{i,j}^{k},{\sum }_{k=1}^{14}{w}_{k}{u}_{i,j}^{k}\right],{\hat{Y}}_{i,j},{\sum }_{k=1}^{14}{w}_{k}\left.{Y}_{i,j}^{k}\right)\right)$$

subject to the constraint ${\sum}_{k}{w}_{k}=1$. The opimisation problem is solved using the SciPy-function minimize with the trustconst-method and an initial guess equal to 1/14 for all *w*_*k*_.

**Fig. 2:**
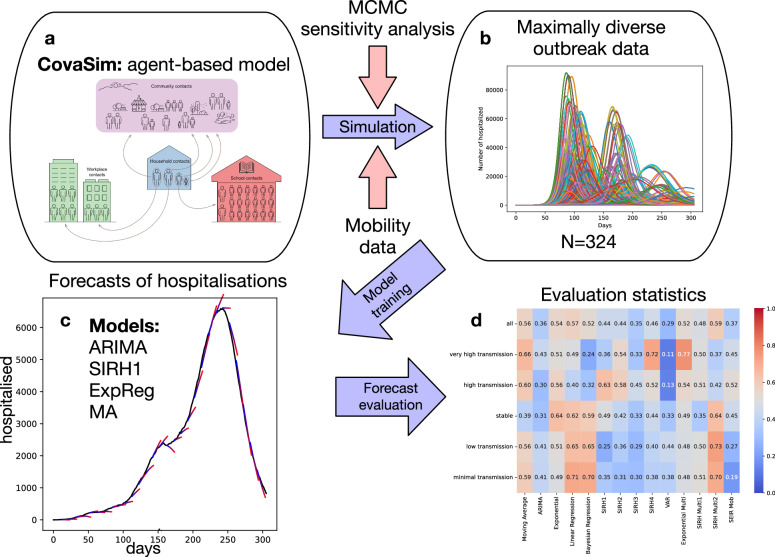
Overview of the methodology. **a** We identified parameters in Covasim, which when varied, gave rise to the most diverse set of outbreak data. **b** Using these parameters we generated 324 different daily time-series of the number of hospitalised patients each X days long. **c** 13 different forecasting models were trained on each time-series and the accuracy of 7 and 14 days ahead predictions was evaluated using RMSE and WIS. **d** Results were aggregated and the models were ranked based on accuracy. Panel a is adapted from ref. ^16^, PLoS Computational Biology, 2021.

**Rank-based Ensemble (EnsRank):** this ensemble changes its weights depending on the current effective number. For each of the five regimes defined in Fig. 2 we pick the five models with the smallest average rank *r*_1_ < *r*_2_ < ⋅ ⋅ < *r*_5_. We now set ${\tilde{w}}_{k}=1/{r}_{k}$ and normalise the weights so that the sum to unity according to

17 $${w}_{k}={\tilde{w}}_{k}/{\sum }_{i=1}^{5}{\tilde{w}}_{i}.$$

The forecast of this ensemble equals

18 $${Y}_{t}^{R}={\sum }_{k=1}^{5}{w}_{k}{Y}_{t}^{k}$$

where the sum runs over the top-ranked models given the current effective reproduction number.

### Ensemble variance

To investigate the relationship between the variation of component model forecasts and ensemble error we calculated the coefficient of variation for all ensemble forecast across all outbreaks. The coefficient of variation is defined according to *CV* = *σ*/*μ* where $\mu =\frac{1}{14}{\sum }_{k=1}^{14}{\hat{Y}}^{k}$ is the mean prediction and $\sigma =\sqrt{\frac{1}{14}{\sum }_{k=1}^{14}{(\mu -{\hat{Y}}^{k})}^{2}}$. The ensemble error was calculated for the median ensemble using the Mean Absolute Percentage Error ${{{\rm{MAPE}}}}_{t}=| {Y}_{t}-{Y}_{t}^{M}| /{Y}_{t}$, where *Y*_*t*_ is the outcome and ${Y}_{t}^{M}$ is the median forecast at time *t*. For the analysis we exclude points where *Y*_*t*_ < 10.

### Estimating the effective reproductive number

The effective reproduction number was estimated using the method described in ref. ^19^, where estimation of *R*_eff_ on a given day only uses historical data. This estimate is known as the instantaneous reproduction number since it only uses data up to the present day. Since the estimate is used as a threshold value to determine which models to include in the adaptive ensemble we only calculate a point estimate and do not calculate the uncertainty of the estimate. We note that this might make the estimate sensitive to fluctuations in incidence. The generation time distribution required for the estimation was assumed to be a gamma distribution. For Covasim data we used a mean of 1/*γ*_*i*_ = 8 days and a standard deviation of 3 days. An example of estimated versus true *R*_eff_ for a Covasim outbreak can be seen in Fig. S5. For the COVID-19 data we used a mean of 4 days and a standard deviation of 3 days^20^.

### Evaluation on real data

Data on the number of hospitalised COVID-19 patients in Sweden during 2020 was obtained from Our World in Data (https://ourworldindata.org/coronavirus). In order to obtain good estimates of the effective reproduction number for COVID-19 during the pandemic in Sweden in 2020, we estimated incidence based on COVID-19 deaths according to the method presented in ref. ^21^. Data on deaths were obtained from the Swedish National Board of Welfare. Mobility data for Sweden was obtained from^5^, which contains data on public transport utilisation from one major Swedish region, which we use as a proxy for mobility at a national level.

Data from the US on patients hospitalised with COVID-19 was obtained from healthdata.gov

(https://healthdata.gov/Hospital/COVID-19-Reported-Patient-Impact-and-Hospital-Capa/g62h-syeh/about_data) and data on daily cases was obtained from Johns Hopkins University: https://github.com/CSSEGISandData/COVID-19.

Data on hospitalisations and daily cases for Czech Republic, Belgium and France was obtained from ECDC.

Mobility data for all countries except Sweden was obtained from Google Community Mobility Reports as the change from baseline in the category Transit stations (https://www.google.com/covid19/mobility/).

Data on hospitalisations, incidence and mobility were compiled into single CSV-files for all countries and are available together with the code at: Zenodo^15^.

## Results

### Overview of the approach

In order to generate synthetic data on hospitalisations from an epidemic we made use of Covasim^16^ an agent-based model of COVID-19 transmission, where disease and transmission parameters can be tuned to represent a wide-range of respiratory tract infections with e.g. different severity profiles, serial interval distributions and fraction of asymptomatics. The transmission dynamics can also be modulated by specifying time-dependent mobility rates.

In the simulation, we consider all cases that are severe and critical to require hospital admission and extract the number of hospitalised patients each day of the simulation. In addition to hospitalisations we also record the incidence, the effective reproduction number and mobility each day.

To generate a maximally diverse set of epidemics we constructed a metric that quantifies the difference between hospitalisation curves and made use of a Markov Chain Monte Carlo-method for identifying the subset of parameters, which when varied give rise to diverse epidemics (see Fig. 2A and ‘Methods’). We identified four parameters (symptomatic-to-severe rate, asymptomatic-to-recovered rate, probability of being symptomatic, probability of becoming severe) that when varied had the largest impact on the epidemic. We ran Covasim with all possible parameters combinations when each parameter was either halved, at baseline or doubled. In addition we considered four different time-dependent mobility rates: seasonal, lockdowns, empirical and constant. This yielded 324 unique epidemics each 300 days long (see Fig. 2B).

On each epidemic we trained 14 different forecast models and every 20 days we made forecasts of the number of hospitalised cases 7 and 14 days ahead (see Fig. 2C). We considered statistical models (e.g. exponential regression), autoregressive models (e.g. ARIMA) and mechanistic models (e.g., an SIRH-model, which is an extension of the SIR-model with a compartment for hospitalised cases). Some models were univariate and only used the past hospitalisations to forecast the future, whereas others were multivariate and used past mobility and incidence for forecasting (e.g. a multivariate exponential regression and a VAR-model). We refer to the ‘Methods’ for a detailed description of all models. The performance of the models was evaluted using, root mean squared error (RMSE), the weighted interval score (WIS) and mean absolute percentage error (MAPE)^22^ (see ‘Methods’). Please note that knowledge about the parameters used in Covasim is only available to a subset of the mechanistic models (see ‘Methods’), and that models are evaluated solely on their ability to forecast the hospitalisation time-series.

### Performance of individual models

**Fig. 3:**
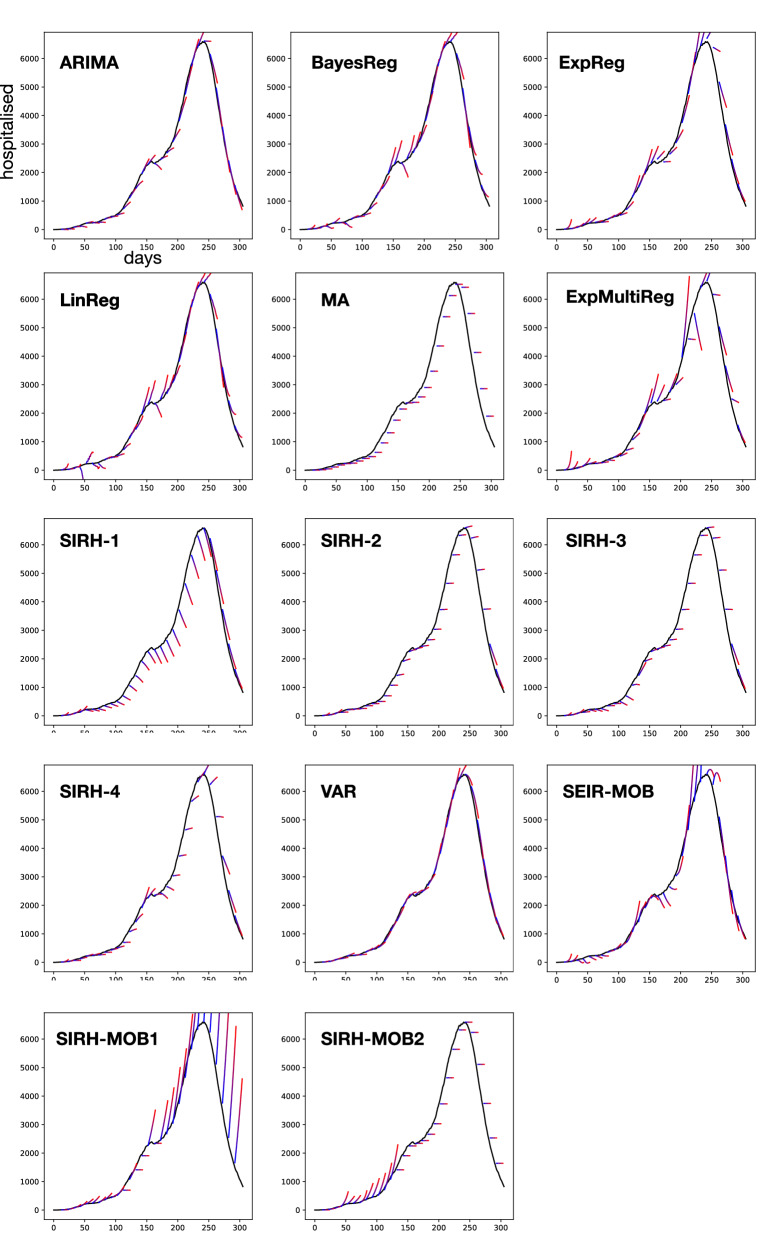
Forecasts of all models on a single example epidemic. Forecast are made every 10 days and stretch 14 days into the future. The colouring of the forecast curves is meant to increase readability and indicates the length of the forecast (blue at day 0 and red at day 14).

Point forecasts of all considered models on an example epidemic are shown in Fig. 3. Forecast are made every 10 days and stretch 1–14 days into the future. Here it can be seen that some models appear to provide accurate point forecast (e.g. the time-series ARIMA- and VAR-models), whereas others such as the SIRH-1 and ExpMultiReg fail to provide as accurate forecasts.

**Fig. 4:**
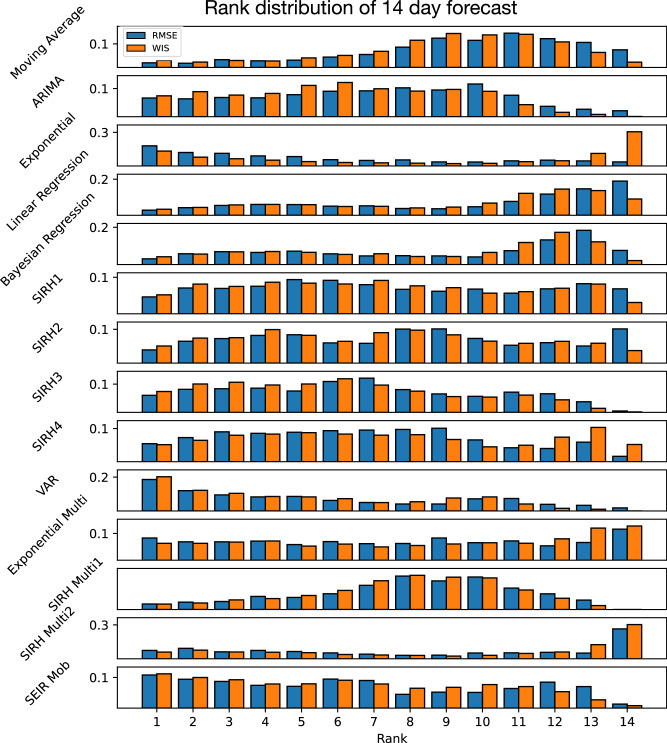
Distribution of rankings of the models for all points for 14-day forecasts with respect to both RMSE and WIS. The average ranks for each model is reported in Table 3.

In order to systematically investigate the accuracy of the models across all 324 epidemics we calculated the RMSE and WIS of each forecast and ranked the models according to their performance. The distribution of ranks based on RMSE and WIS is shown in Fig. 4, where height of the bars correspond to the fraction of times each model achieved the corresponding rank. The Moving Average (MA) model represents our baseline model and forecasts the average number of hospitalised cases in 7 day period prior to the day of forecast. As expected this model performs poorly with respect to both the WIS and RMSE metrics, although it does perform best in a small fraction of cases. In fact for most models the rank distribution with respect to WIS and RMSE are similar, the exception being the Exponential Regression model. This discrepancy arises because the ExpReg-model provides accurate point predictions, but (at times) excessively wide prediction intervals, which increases the WIS and places the model at the bottom of the ranking. The rank distribution for 7 day forecast is similar (see Supplementary Fig. 1).

The rank distribution can be summarised by considering the average ranks of the models, which is shown in Table 3. Here we have also normalised the ranks so that 0 corresponds to the best possible rank and 1 to the worst. To begin with note that the Moving Average (MA) model has the worst average normalised rank in all cases except for WIS at 14 days where Linear Regression and SIRH-Multi2 perform worse. We can see that the top performing models with respect to RMSE for 7 days is VAR, ExpReg and ARIMA, and for 14 days we have ExpReg, VAR and SEIR-Mob. If we instead consider WIS the top performing models for 7 days are VAR, ARIMA and SIRH3, and for 14 days forecasts: VAR, SIRH3 and ARIMA.

**Table 3:** Average normalised rank of all 14 models for 7- and 14-day forecasts with respect to RMSE and WIS

Model	RMSE (7 days)	WIS (7 days)	RMSE (14 days)	WIS (14 days)
Moving Average	0.69	0.62	0.61	0.56
ARIMA	0.37	0.31	0.43	0.36
Exponential	0.34	0.55	**0.33**	0.54
Linear Regression	0.50	0.47	0.60	0.57
Bayesian Regression	0.44	0.42	0.55	0.52
SIRH1	0.50	0.47	0.47	0.44
SIRH2	0.53	0.47	0.48	0.44
SIRH3	0.43	0.36	0.40	0.35
SIRH4	0.45	0.51	0.41	0.46
VAR	**0.29**	**0.26**	0.33	**0.29**
Exponential Multi	0.49	0.54	0.48	0.52
SIRH Multi1	0.55	0.53	0.50	0.48
SIRH Multi2	0.53	0.61	0.52	0.59
SEIR Mob	0.41	0.38	0.40	0.37

The best model (lowest average rank) is highlighted in bold in each column.

It is worth noting that the top models contain both statistical and mechanistic models. If we consider the change in normalised rank going from 7 to 14 days we see that all models that have an autoregressive structure (ARIMA, LinReg, BayesReg and VAR) perform worse for longer forecast horizons. This is true for both WIS and RMSE. In contrast, all mechanistic models perform better at 14 days compared to 7 days (for both RMSE and WIS). The latter also holds true for the Moving Average-model and the two exponential regression models.

Comparing the the SIRH-models we note that accuracy does not appear to be related to the amount of information about the disease available to the forecaster. SIRH1, which has the rate of recovery for regular and severe/hospitalised cases fixed at the true values only performed marginally better in terms of WIS and worse in terms of RMSE when compared to SIRH4, in which the above rates are estimated from data.

The average normalised rank is a relative measure of performance and in order to evaluate the accuracy of the forecasts we also measured the trimmed Mean Absolute Percentage Error (MAPE) across all outbreaks (see ‘Methods’), which is shown in Table 4. In this comparison the Exponential-model performs best for both forecast horizons indicating a consistency across different forecasts which is not achieved by e.g. the VAR-model, which has a low average normalised rank, but a comparatively high MAPE.

**Table 4:** Trimmed mean absolute percentage error (MAPE) for all models and 7- and 14-day forecasts

Model	MAPE (7 days)	MAPE (14 days)
Moving Average	0.55	1.00
ARIMA	0.32	1.35
Exponential	**0.17**	**0.39**
Linear Regression	0.76	5.45
Bayesian Regression	0.47	3.12
SIRH1	0.27	0.50
SIRH2	0.37	0.57
SIRH3	0.24	0.47
SIRH4	0.33	0.54
VAR	0.31	1.10
Exponential Multi	0.37	0.96
SIRH Multi1	0.37	0.78
SIRH Multi2	1.35	4.18
SEIR Mob	0.21	0.41

The model with the lowest error is highlighted in bold in each column.

**Fig. 5:**
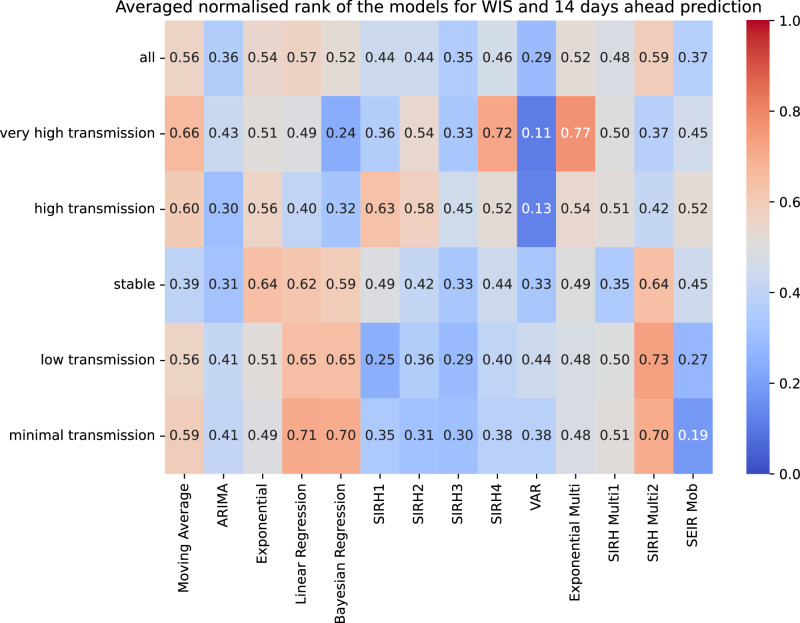
Heatmap of average normalised model rank based on WIS for 14-day forecasts. The day of forecast is classified according to the current effective reproduction number according to: minimal transmission (*R*_eff_ < 0.5), low transmission (0. 5 ≤ *R*_eff_ < 0.8), stable (0. 8 ≤ *R*_eff_ < 1.2), high transmission (1.2 ≤ *R*_eff_ < 3) and very high transmission *R*_eff_ ≥ 3.

We then went on to stratify the average normalised ranks based on the characteristic of the epidemic in terms of the effective reproductive number *R*_eff_ at the day the forecast was made. We consider five different classes ranging from minimal transmission (*R*_eff_ < 0.5), low transmission (0.5 ≤ *R*_eff_ < 0.8) to stable (0.8 ≤ *R*_eff_ < 1.2), high transmission (1.2 ≤ *R*_eff_ < 3) and very high transmission *R*_eff_≥3. The results can be seen in Fig. 5 which shows the average normalised rank using WIS at 14 days. It is clear that model performance in general is quite heterogeneous with respect to *R*_eff_. For example, the VAR-model performs very well at times of high and very high transmission (average rank of 0.13 and 0.11 respectively), but considerably worse at low transmission (average rank 0.44), where it is outperformed by six other models. On the other hand the SEIR-Mob model is the best model during minimal transmission (average rank 0.19), but is outperformed by seven other models during high transmission (results for WIS 7 days and RMSE for 7 and 14 day forecasts are similar, see Supplementary Fig. 2). This suggests that knowledge about the current status of the epidemic can inform model choice, a fact we will return to when constructing ensemble models (also see the Discussion concerning the difficulties of making use of this information in a real-world setting).

**Fig. 6:**
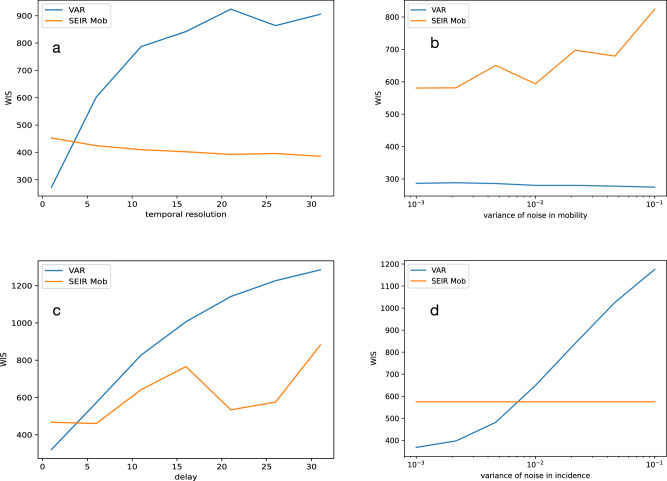
Performance of the VAR- and SEIR Mob-model under perturbations. Note that lower WIS corresponds to higher accuracy and the average WIS of the baseline MA-model equals 1475. **a** The temporal resolution is lowered so that it changes every *x* days, **b** white noise is added to the mobility data with a certain variance, **c** The mobility and incidence data are delayed with *x* days and **d** noise from a Poisson-Gamma mixture with varying variance is added to the incidence data. See ‘Methods’ for details concerning the perturbations.

As noted earlier, some models make use of multivariate data to make forecasts of future hospitalisations. These additional data streams in terms of incidence and mobility are provided to the models at the same daily resolution as the hospitalisation data and without any noise or delay. In a sense, this makes for an unfair comparison between univariate and multivariate models since incidence and mobility data rarely is available at such high temporal resolution and without any delay or noise. To investigate the impact of such limitations on the data streams we considered four distinct perturbations: (i) the temporal resolution of the data streams is lowered (ii) uncorrelated noise is added to the mobility data with a certain variance (iii) the mobility and incidence data are delayed and (iv) uncorrelated noise with a certain variance is added to the incidence data (see ‘Methods’ for details concerning the perturbations). Using the perturbed data streams we generated predictions for the multivariate VAR and SEIR Mob-models, which both performed well compared to the other models, and measured the WIS averaged across all outbreaks and predictions. The result can be seen in Fig. 6. In panel A we see that for the VAR-model, which uses both incidence and mobility, the performance decreases (corresponding to an increase in WIS) when the resolution is decreased. For reference the average WIS of the Moving Average model equals 1475, which is above the WIS of the VAR-model for all resolutions. In contrast, the SEIR-Mob model, which only uses mobility data, performs even better (i.e. lower WIS) when the resolution is increased to 20 days, beyond which performance flattens out. This implies that the mechanistic SEIR-Mob model performs better when the mobility data is updated less frequently (see Supplementary Fig. 3 for an outbreak where this occurs). Adding noise to the mobility data has little effect on performance of both models (see Fig. 6B), whereas introducing noise to the incidence data clearly affects WIS for the VAR-model (see Fig. 6D) (the SEIR model is unaffected since it does not use incidence data). Lastly, delaying the data streams reduces performance of both models, with the VAR-model being more sensitive (see Fig. 6C).

### Ensemble models

It has been established that ensemble forecasts typically outperform component models in terms of performance^23^. We investigate this observation in the context of our large-scale synthetic dataset and consider the following types of ensembles:

- Average Ensemble (EnsAvg): this ensemble takes an unweighted mean of all component models to calculate the point prediction and prediction intervals^3^.
- Median Ensemble (EnsMedian): this ensemble uses the median of all point predictions and prediction intervals^24^.
- Regression Ensemble (EnsReg): this ensemble takes a weighted mean of the component point predictions and prediction intervals plus an intercept. The weights and intercept were obtained using linear regression where the component model forecasts are covariates and the actual hospitalisation is the target variable.
- RMSE-optimised Ensemble (EnsRMSE): same as EnsReg with the difference that the weights are constrained to sum to unity and there is no intercept^14^.
- WIS-optimised Ensemble (EnsWIS): same as EnsRMSE but here the weights are selected to minimise the WIS of the ensemble forecast. Again the weights sum to unity^14^.
- Rank-based Ensemble (EnsRank): this ensemble changes its weights depending on the current effective reproductive number. For each of the five regimes defined in Fig. 5 we pick the five models with the smallest average rank. The weights of the models are set to the inverse of the model rank, and lastly the weights are normalised to sum to unity, similar to what is done in ref. ^23^.

**Fig. 7:**
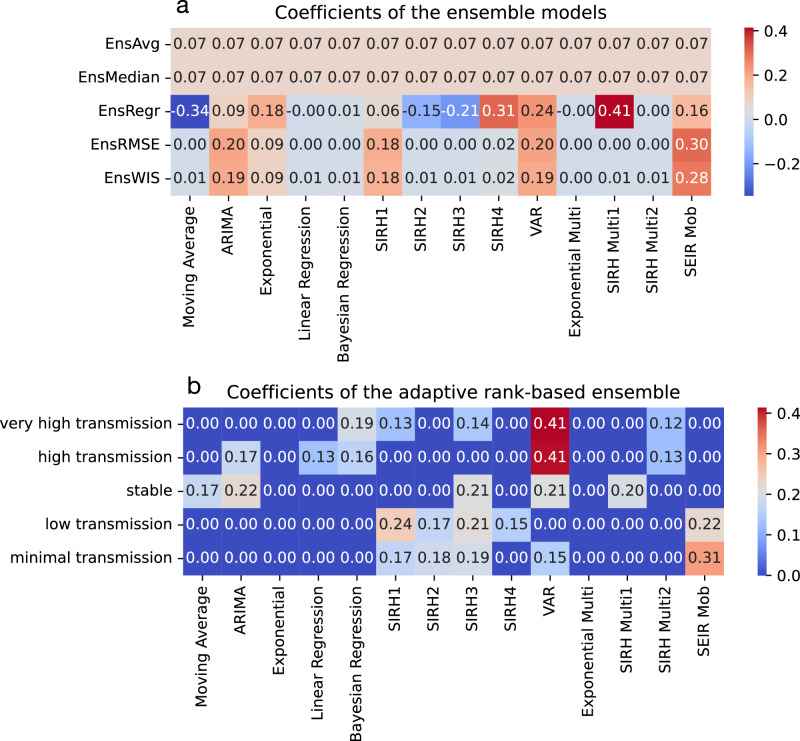
Construction of ensemble forecasts. **a** The weights of component model predictions in the ensembles. Note that for the Median Ensemble (EnsMedian) the point prediction is given as the median of the model predictions. This also applies to the quantiles of the EnsMedian-prediction. **b** The weights in the rank-based adaptive ensemble that are choosen depending on the current effective reproductive number.

The model weights were obtained by splitting the epidemics into a training and evaluation set and calibrating the weights on the training set. The resulting model weights are shown in Fig. 7.

**Fig. 8:**
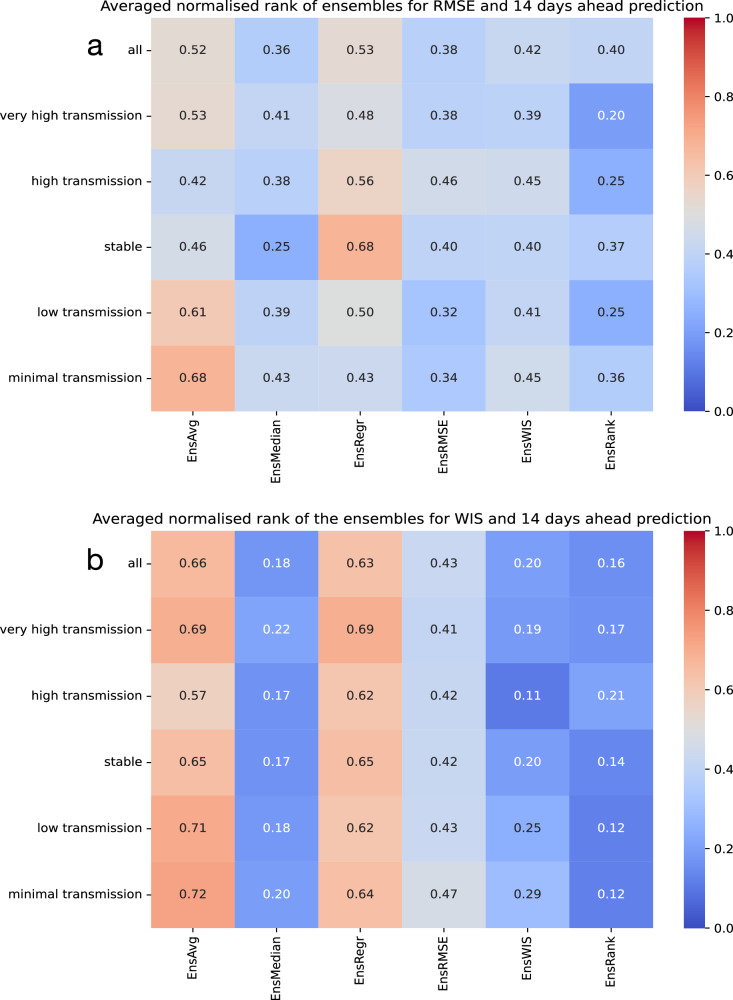
Normalised ensemble ranks. The average normalised ensemble rank as compared to both component models and the six ensembles for (**a**) RMSE and **b** WIS.

We then evaluated the performance of the ensembles on the evaluation set with respect to both WIS and RMSE. The average normalised rank of the ensembles when compared to all models (in total 20 models, 14 component models plus 6 ensembles) is shown in Fig. 8. In terms of RMSE-performance the ensembles do not outperform the component models when considering all points, but do so for some types of points, e.g. EnsRank has the lowest average normalised rank for ‘low transmission’ and EnsMedian has the lowest average normalised rank when transmission is ‘stable’. However, for WIS-performance the picture is different. Here, three ensembles, EnsMedian, EnsWIS and EnsRank, clearly outperform all component models and other ensembles. EnsRank has the lowest overall rank among the ensembles for all transmission regimes except ‘high transmission’, where EnsMedian has the lowest average rank.

The performance of EnsRank (in contrast to EnsMedian) is dependent on weights obtained by training the ensemble on a fraction of the Covasim outbreaks. We therefore investigated how the fraction of outbreaks used in training affects the performance of EnsRank, and the results show that performance is unaffected by the fraction of training outbreaks down to approximately 15% below which performance rapidly degrades (see Supplementary Fig. 4).

EnsRank is also different from the other ensembles since it relies on the current value of the effective reproductive number. In the above analysis we obtained the effective reproductive number from the Covasim simulation, but in a real setting one would need to estimate *R*_eff_ from available data. We mimicked this situation by estimating *R*_eff_ from incidence data from Covasim (see Supplementary Fig. 5). The normalised ranks obtained using estimated *R*_eff_ are very similar and see that EnsRank still obtains the lowest average normalised rank for WIS (see Supplementary Fig. 6).

In situations where component models in an ensemble agree it is reasonable to think that the ensemble should be more accurate compared to a situation where component predictions diverge. To investigate this hypothesis we measured the accuracy of the median ensemble in terms of the mean absolute percentage error (MAPE) between the median ensemble forecast and the outcome. The level of agreement between the component forecasts that constitute the ensemble was quantified using the coefficient of variation (CV) across the component forecast within the ensemble. CV is calculated as the sample standard deviation divided by the sample mean and is a normalised measure of variation within a sample. The sample here refers to all component forecasts made at a specific time *t*. We then paired up the CVs calculated at day *t* with the forecast errors calculated at day *t* + 7.

**Fig. 9:**
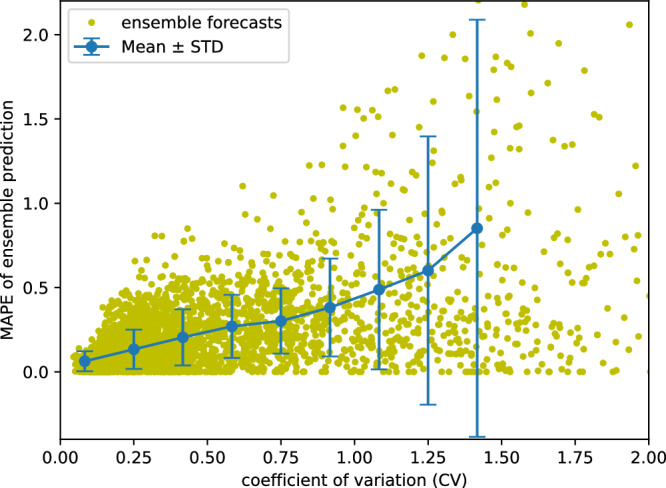
The spread-skill relationship. Each point corresponds to a single 7-day forecast of the median ensemble, where the x-coordinate is equal to the coefficient of variation (CV) of the component model predictions and the y-coordinate is equal to the error (MAPE) of the forecast. The solid line shows the mean MAPE in each bin and the error bars correspond to one standard deviation (see Supplementary Fig. 7 for the corresponding plot for 14 day forecasts).

Each such pair for every forecast is plotted in Fig. 9 with CV on the x-axis and MAPE on the y-axis, together with the mean and standard deviation of the MAPE in each bin (the corresponding figure for 14-day forecast is Supplementary Fig. 7). From the plot it is clear that a small CV implies a small MAPE, whereas for large CVs the MAPE can take a range of values. Although the data has a strong heteroscedastic trend we observe an increased mean MAPE as the CV of the ensemble increases. This implies that the CV calculated at day *t* of the ensemble is predictive of future error of the ensemble forecast obtained at day *t* + 7 and *t* + 14.

By assuming a simple statistical model for the relationship between spread and error an upper bound on the correlation coefficient between the variables can be obtained^25^. In our data we find that for CV in the range 0 < CV < 2 the correlation coefficient between CV and MAPE-error equals 0.51, whereas the theoretical upper bound, which assumes an unbiased ensemble, equals approximately 0.6.

### Evaluation on real data

To validate our findings concerning the performance of the different ensemble methods we evaluated all component models and the ensembles on hospitalisation data for COVID-19 during 2020 in five different countries (Sweden, France, Czech republic, Belgium and the US). In order to imitate the situation during a real outbreak we train the component models on increasing amounts of hospitalisation data while retaining the ensemble weights obtained by training on the synthetic data. The rationale for this is that while ensemble weights can be obtained by training on synthetic data (assuming that the synthetic data is diverse enough to capture the dynamics of the ongoing outbreak) the individual models need to calibrated to the ongoing outbreak with its specific disease characteristics.

**Fig. 10:**
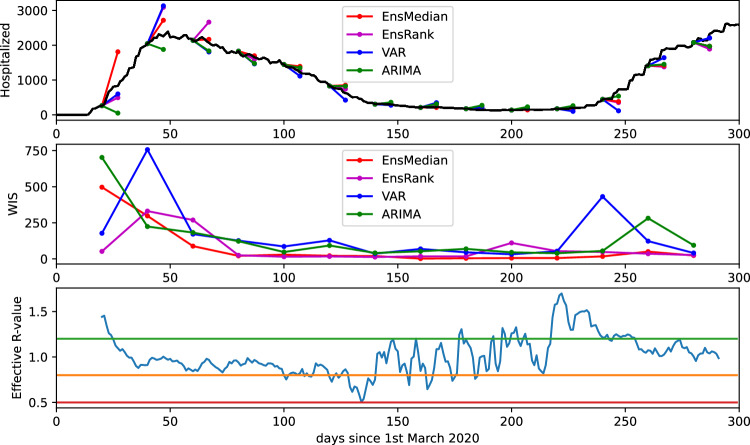
Visualisation of selected component model and ensembles 7-day forecasts for COVID-19 data from Sweden during 2020. Top panel shows point predictions from the best performing ensembles (median- and adaptive rank-based) and component models (VAR and ARIMA). The middle panel shows the weighted interval score (WIS) of the ensembles and models calculated from prediction intervals for each forecast. The lower panel shows the estimated effective reproduction value. The solid lines correspond to break points for the adaptive rank-based ensemble. The red line corresponds to *R*_eff_ = 0.5, the orange line to *R*_eff_ = 0.8 and the green line to *R*_eff_ = 1.2.

On the COVID-19 data we trained all 14 forecast models and every 20 days we made forecasts of the number of hospitalised cases 7 and 14 days ahead. We calculated the WIS, RMSE and MAPE for all forecasts. The resulting forecasts and WIS from a subset of forecasts for Sweden are shown in Fig. 10 together with the instantaneous reproductive number estimated from incidence data^19^, which is used by the rank-based ensemble (plots for the remaining countries can be seen in Supplementary Fig. 8). The weights in EnsRank were the same as for the synthetic evaluation and were not adjusted for the real data. For the Swedish dataset we see that the ensemble forecasts achieve lower WIS and EnsRank achieves a slightly lower average WIS compared to EnsMedian (73.8 vs 77.4).

The performance on all five datasets of all component models and ensembles is summarised in Table 5, which shows the relative rank based on RMSE and WIS for 7- and 14-day forecasts, and Table 6, which shows the trimmed MAPE for 7- and 14-day forecasts. We note that EnsMedian has the lowest normalised rank except for RMSE (7-days) where EnsRank performs best. Despite EnsMedian exhibiting a lower average rank, we note that EnsRank outperforms EnsMedian in 27% of all 7-day forecasts and 40% of all 14-day forecasts. In terms of MAPE EnsMedian performs best for both forecast horizons. Comparing forecast accuracy with the baseline Moving Average-model we see that 15/19 ≈ 78% of models perform better than MA for 7-day forecasts, whereas for 14-days the corresponding fraction drops to 2/19 ≈ 11%.

**Table 5:** Average normalised rank of all 14 component models and 5 ensembles for 7- and 14-day forecasts with respect to RMSE and WIS

Model	RMSE (7 days)	WIS (7 days)	RMSE (14 days)	WIS (14 days)
Moving Average	0.60	0.51	0.51	0.46
ARIMA	0.43	0.39	0.47	0.41
Exponential	0.43	0.70	0.40	0.68
Linear Regression	0.40	0.40	0.43	0.43
Bayesian Regression	0.38	0.35	0.45	0.44
SIRH1	0.66	0.60	0.66	0.62
SIRH2	0.46	0.44	0.44	0.42
SIRH3	0.55	0.51	0.55	0.52
SIRH4	0.49	0.58	0.49	0.57
VAR	0.49	0.44	0.47	0.45
Exponential Multi	0.41	0.50	0.38	0.49
SIRH Multi1	0.50	0.49	0.49	0.49
SIRH Multi2	0.70	0.71	0.70	0.73
SEIR Mob	0.48	0.51	0.45	0.48
EnsAvg	0.45	0.69	0.51	0.64
EnsMedian	0.35	**0.11**	**0.33**	**0.10**
EnsRegr	0.63	0.69	0.55	0.66
EnsRMSE	0.36	0.51	0.38	0.46
EnsWIS	0.39	0.20	0.41	0.20
EnsRank	**0.34**	0.19	0.43	0.24

The lowest rank in each column is marked in bold.

**Table 6:** Trimmed MAPE across forecast for all countries

Model	MAPE (7 days)	MAPE (14 days)
Moving Average	0.26	0.38
ARIMA	0.22	0.48
Exponential	0.22	0.49
Linear Regression	0.21	0.90
Bayesian Regression	0.16	0.51
SIRH1	0.27	0.48
SIRH2	0.18	0.31
SIRH3	0.25	0.53
SIRH4	0.21	0.48
VAR	0.22	0.44
Exponential Multi	0.19	0.40
SIRH Multi1	0.28	0.72
SIRH Multi2	0.93	3.43
SEIR Mob	0.21	0.44
EnsAvg	0.22	1.45
EnsMedian	**0.14**	**0.27**
EnsRegr	0.67	1.28
EnsRMSE	0.15	0.39
EnsWIS	0.16	0.41
EnsRank	0.15	0.56

The lowest value in each column is marked in bold.

## Discussion

We have investigated the ability of a wide array of forecasting models to predict the number of hospitalised cases of a hypothetical respiratory infectious disease. A diverse set of outbreak data was generated using Covasim, an established agent-based model of COVID-19 transmission, whose parameters were adjusted to generate a maximally diverse set of hospitalisation curves (see Fig. 2B).

We considered 14 forecasting models that were either autoregressive, statistical or mechanistic and were univariate or multivariate (made use of multiple data types for prediction). The accuracy of the models was evaluated using RMSE and MAPE (for point predictions) and WIS (for probabilistic predictions) for 7- and 14-day forecasts. For each forecast we ranked the models according to their RMSE/WIS with the first ranked model having the lowest error. The resulting rank distributions (Fig. 4) based on all epidemics and forecasts are highly heterogeneous were no model consistently outperforms the others (points with no or very low hospitalisations are excluded in order to avoid bias, see ‘Methods’ for details). Similar findings have been made by forecast hubs during the COVID-19 pandemic^3^.

In fact all models are at some point both the worst and best model. However, there are individual differences where some models have distributions shifted towards higher rank (e.g. Bayesian regression), whereas others are shifted towards low rank (e.g. the VAR-model). The rank distributions with respect to RMSE and WIS are in general similar, but Exponential regression is an exception with a distribution which is shifted towards lower rank with RMSE and higher rank with WIS. The reason behind this is that WIS punishes forecast with excessive prediction intervals, which the Exponential regression model at times produces. The Exponential regression model also exhibits the lowest MAPE for both 7 and 14 day forecasts again highlighting the accuracy of its point predictions.

Taking the average of the rank distributions and normalising (see Table 3) we notice that almost all models outperform the Moving Average-model, which serves as a baseline model, the exception being Linear Regression and the SIRH-Multi2, which perform worse with respect to WIS at 14 day forecasts. The model performance relative to the baseline model found in this study is better than the one reported in ref. ^3^, which possibly is related to the larger and more diverse dataset used for evaluation.

Going from 7 to 14 day forecasts we note that all autoregressive models decrease their relative accuracy with respect to both RMSE and WIS, whereas the mechanistic models improve their accuracy. The two exponential regression models also show a slight improvement for longer forecasts. The relative improvement of mechanistic models is in line with previous studies that have shown that capturing transmission dynamics in the model structure improves long-term accuracy^26^. In terms MAPE all models show decreased performance going from 7 to 14 days forecasts, which is to be expected. All mechanistic models except SIRH-Multi2 show a less than twofold increase in MAPE, while the autoregressive and statistical models exhibit considerably larger deterioration in accuracy, e.g. the VAR-model which has a 3.5-fold higher MAPE for the 14 day forecast.

The heterogeneity of performance becomes even more evident when we stratified the rank based on the effective reproduction value at the time of forecast (see Fig. 5). From this it can be seen that the autoregressive models in general perform better during high/very high transmission compared to low/minimal.

This information could, after proper validation, guide the use of different forecast models during an ongoing pandemic or epidemic. Given a set of forecast model at the disposal of a decision-maker it would be possible to evaluate the performance of those models on synthetic data that is representative of the ongoing outbreak, and stratify the accuracy based on effective reproductive number. The results could then inform decision-makers when considering multiple forecasts. However, this approach has several potential drawbacks that require further research, e.g. how does uncertainty and lags in reported data influence the estimation of the effective reproductive number and the feasability of producing timely forecast. Another important question to resolve is how diverse the synthetic data should be to best inform the choice of forecast at different stages in the outbreak.

As we have shown, the stratification can also be used to form an adaptive rank-based ensemble, where the weights of component models are adjusted depending on the instantaneous reproduction number. This rank-based ensemble outperforms all other ensemble methods on synthetic data (see Fig. 8), although the median ensemble and WIS-optimised ensemble show similar performance. However, the good performance of the median ensemble, which has been reported previously^14^, together with its simplicity makes it a strong candidate for producing forecasts. In particular, since it requires no adjustment of model weights based on historical data.

When investigating the effect of perturbations of the mobility and incidence we saw that the mechanistic SEIR Mob-model was more robust compared to the auto-regressive VAR-model (see Fig. 6). Somewhat surprisingly the SEIR Mob-model performed better when the temporal resolution was lowered. The reason for this has not been established, but we hypothesise that mobility trends on longer time scales are sufficient to describe the transmission dynamics, and at the same time it might be easier to train the model when the mobility changes less frequently. This is a topic which merits further investigation.

Evaluation of the component models and ensembles on real data from the first year of the COVID-19 pandemic showed that rank-based ensemble performed well also in this context, but the median ensemble had the lowest average normalised rank for three out of four performance measures (see Table 5). However, the rank-based ensemble outperforms the median in 27% of all 7-day forecasts and 40% of all 14-day forecasts, which merits further investigation into this novel type of adaptive ensembles. Looking at the component models almost all models have an average normalised rank in the range 0.4-0.6, meaning that no model stands out as particularly high or low performing. A similar picture can be seen in the MAPE evaluation (see Table 6), where many models perform similar or worse to the baseline Moving Average-model. For 14-days forecasts only one component model outperforms the Moving Average, which is the SIRH2-model. Despite this, the median ensemble achieves a considerably lower MAPE compared to the Moving Average (0.27 compared to 0.38)

A benefit of our approach with a large-scale synthetic, yet realistic, data set is that it is possible to observe statistical trends not observable in a single epidemic. One notable example of this is the relation between the coefficient of variation (CV) of component forecasts within an ensemble and the future error of the ensemble forecast (see Fig. 9). That ensemble CV is indicative of forecast error has previously been observed in meteorology, where ensembles are used to account for both uncertainty in initial condition and model structure^27^. In meteorology, this is known as the spread-skill relationship and has been shown to be informative about the expected quality of the forecast. However, it should be noted that the relationship between CV and error is highly heteroscedastic and further analysis and validation is required before the CV can be used operationally to assign confidence to ensemble forecasts. A similar relationship was observed by Shaman & Karspeck^28^ who made use of an ensemble adjustment Kalman filter to model influenza epidemics. They used a single model for disease transmission, and the ensemble was formed by multiple instances of the model with different states and parameter values. In that context they showed that that the logarithm of the ensemble variance was predictive of the ability of the ensemble to forecast the timing of the peak of the outbreak. This is similar to our results, but yet distinct since we consider a multi-model ensemble with 14 different component models.

This study has several limitations. To begin with we consider synthetic data generated using an agent-based model of respiratory disease transmission. Although this model accounts for many of the processes involved in disease transmission it might still miss out on important characteristics of real data. In addition we assumed perfect access to hospital and mobility data without any delays or misreporting. In realistic settings there is typically a reporting delay which reduces the effective forecasting horizon. Reporting delays can be handled using nowcasting methods^29^, but this introduces additional uncertainty in recent data.

Secondly, we only consider a limited set of forecast models. Some of these models have been used during the COVID-19 pandemic to predict hospital admission^5,30–32^, but our study does not include other commonly used models such as age-structured compartmental models, machine learning models and agent-based models. Some of the models we consider make use of additional data streams in terms of incidence and mobility, but there are other data sources that can be informative when forecasting hospitalisations, e.g. telenursing calls^33^ and waste-water data^34^. When additional data streams are used for the sake of prediction we assume that they either remain constant (for Exponential Multi) or make a linear extrapolation for future values based on historical data (for SIRH-Multi and SEIR-Mob). This represents simple forms of extrapolation into the future, and it also possible to account for fluctuations and uncertainty in the data streams when extrapolating^35^. The use of real mobility data (from the COVID-19 outbreak in Sweden) to modulate the contact intensity in Covasim increases the realism of the synthetic data, but it also introduces a certain level of bias when evaluating performance on the COVID-19 pandemic since Sweden was one of the six countries which was included in the evaluation. However, we note that the median ensemble has a lower average WIS for Czechia compared to Sweden (55.7 vs. 77.4) suggesting that the bias introduced by the Swedish data is limited.

Our study focused on hospitalised cases as the target variable, but there are a number of other variables that are of interest to decision-makers during an epidemic. For example, forecasting incidence can be useful since changes in incidence typically precede changes in hospitalisation, thus providing an early warning signal. Also, forecasting mortality has been considered useful since it provides an estimate of coming severity of an outbreak.

The conclusions drawn from the results concerning different ensemble methods and the spread-skill relationship are based on the component models considered in this study. In order to strengthen those conclusion one would like to carry out similar studies with respect to both different component models and target data sets. Optimally this would be performed in a prospective setting on real data. Also, our results are limited to short-term forecasting and it would be interesting to see if similar results concerning ensembles are obtained for longer term forecasts or even scenario projections.

Lastly, the results from this study are limited to emergent outbreaks in a completely susceptible population, and therefore do not apply to seasonal outbreaks such as influenza. It would be interesting to investigate how well a rank-based ensemble performs in such a setting. A natural starting point for such a study would be the FluSight challenge dataset, which contains forecasts of hospitalisations due to influenza^12^.

In conclusion, this study, which is based on synthetic outbreak data, shows that forecast model performance is heterogeneous with respect to different outbreaks and disease transmission regime. We have shown that this information can be harnessed to form an adaptive rank-based ensemble, which outperforms traditional ensemble types. In future work we plan to investigate how well the rank-based ensemble generalises to other contexts and how the performance depends on the amount of training data. In addition, more research is needed to determine the optimal number and location of the thresholds of the effective reproductive number. The inclusion of other ensemble methods such as Bayesian model averaging^36^ and linear opinion pools^37^ would also be interesting. The spread-skill relationship of ensembles that we have uncovered also requires more investigation, in particular in the context of other component models and datasets.

### Ethics approval

This study used only publicly available, secondary datasets from Our World in Data, healthdata.gov, the Johns Hopkins University Centre for Systems Science and Engineering COVID-19 GitHub repository, the European Centre for Disease Prevention and Control, Google COVID-19 Community Mobility Reports, and the Swedish National Board of Health and Welfare. All analyses were conducted using aggregate population-level data. The authors did not recruit participants, collect new data from individuals, or access identifiable individual-level information. Google Community Mobility Reports are released as aggregated and anonymised metrics, and the remaining sources provide public-use or official aggregate surveillance/statistical data. Accordingly, ethical approval and informed consent were not required.

## Supplementary information

Transparent Peer Review file

Supplemental Information

## Data Availability

All data used in this study is available at: Zenodo^15^.

## Code availability

All code used in this study is available at: Zenodo^15^.

## References

[1] Lutz, C. S. et al. Applying infectious disease forecasting to public health: A path forward using influenza forecasting examples. *BMC Public Health* **19**, 1–12 (2019). 31823751 10.1186/s12889-019-7966-8 PMC6902553

[2] Runge, M. C. et al. Scenario design for infectious disease projections: Integrating concepts from decision analysis and experimental design. *Epidemics* **47**, 100775 (2024). 38838462 10.1016/j.epidem.2024.100775 PMC12444779

[3] Cramer, E. Y. et al. Evaluation of individual and ensemble probabilistic forecasts of COVID-19 mortality in the United States. *Proc. Natl. Acad. Sci.* **119**, e2113561119 (2022). 35394862 10.1073/pnas.2113561119 PMC9169655

[4] Fox, S. J. et al. Real-time pandemic surveillance using hospital admissions and mobility data. *Proc. Natl. Acad. Sci.* **119**, e2111870119 (2022). 35105729 10.1073/pnas.2111870119 PMC8851544

[5] Gerlee, P. et al. Predicting regional COVID-19 hospital admissions in Sweden using mobility data. *Sci. Rep.* **11**, 24171 (2021). 34921175 10.1038/s41598-021-03499-y PMC8683437

[6] Kobres, P.-Y. et al. A systematic review and evaluation of Zika virus forecasting and prediction research during a public health emergency of international concern. *PLoS Negl. Trop. Dis.* **13**, e0007451 (2019). 31584946 10.1371/journal.pntd.0007451 PMC6805005

[7] Munday, J. D., Rosello, A., Edmunds, W. J. & Funk, S. Forecasting the spatial spread of an Ebola epidemic in real-time: Comparing predictions of mathematical models and experts. *eLife* **13**, RP98005 (2024). 10.7554/eLife.98005 PMC12513718 41070719

[8] Reich, N. G. et al. A collaborative multiyear, multimodel assessment of seasonal influenza forecasting in the United States. *Proc. Natl. Acad. Sci.* **116**, 3146–3154 (2019). 10.1073/pnas.1812594116 PMC6386665 30647115

[9] Nixon, K. et al. An evaluation of prospective COVID-19 modelling studies in the USA: From data to science translation. *Lancet Digital Health* **4**, e738–e747 (2022). 36150782 10.1016/S2589-7500(22)00148-0 PMC9489063

[10] Reich, N. G. et al. Collaborative hubs: making the most of predictive epidemic modeling. *Am. J. Public Health* **112**, 839–842 (2022). 10.2105/AJPH.2022.306831 PMC9137029 35420897

[11] Cramer, E. Y. et al. The United States COVID-19 Forecast Hub Dataset. *medRxiv*. https://www.medrxiv.org/content/10.1101/2021.11.04.21265886v1 (2021). 10.1038/s41597-022-01517-w PMC9342845 35915104

[12] Mathis, S. M. et al. Evaluation of flusight influenza forecasting in the 2021–22 and 2022–23 seasons with a new target laboratory-confirmed influenza hospitalizations. *Nat. Commun.* **15**, 6289 (2024). 39060259 10.1038/s41467-024-50601-9 PMC11282251

[13] Ray, E. L. et al. Challenges in training ensembles to forecast Covid-19 cases and deaths in the United States. *Int. Inst. Forecast.* https://forecasters.org/blog/2021/04/09/challenges-in-training-ensembles-to-forecast-covid-19-cases-and-deaths-in-the-united-states/ (2021).

[14] Brooks, L. C. et al. Comparing ensemble approaches for short-term probabilistic Covid-19 forecasts in the US. *Int. Inst. Forecast.* **39** (2020).

[15] Béchade, G., Lundh, T. & Gerlee, P. Evaluation of respiratory disease hospitalisation forecasts using synthetic outbreak data. 10.5281/zenodo.20718137. Accessed: 2026-06-16 (2026). 10.1038/s43856-026-01802-4 PMC13421584 42527441

[16] Kerr, C. C. et al. Covasim: An agent-based model of Covid-19 dynamics and interventions. *PLOS Comput. Biol.* **17**, e1009149 (2021). 34310589 10.1371/journal.pcbi.1009149 PMC8341708

[17] Boyd, S., Diaconis, P., Parrilo, P. & Xiao, L. Fastest mixing Markov chain on graphs with symmetries. *SIAM J. Optim.* **20**, 792–819 (2009).

[18] Doob, J. L. The limiting distributions of certain statistics. * Ann. Math. Stat.* **6**, 160–169 (1935).

[19] Cori, A., Ferguson, N. M., Fraser, C. & Cauchemez, S. A new framework and software to estimate time-varying reproduction numbers during epidemics. *Am. J. Epidemiol.* **178**, 1505–1512 (2013). 24043437 10.1093/aje/kwt133 PMC3816335

[20] Knight, J. & Mishra, S. Estimating effective reproduction number using generation time versus serial interval, with application to COVID-19 in the Greater Toronto Area, Canada. *Infect. Dis. Model.* **5**, 889–896 (2020). 33163739 10.1016/j.idm.2020.10.009 PMC7604055

[21] Wacker, A. et al. Estimating the sars-cov-2 infected population fraction and the infection-to-fatality ratio: A data-driven case study based on Swedish time series data. *Sci. Rep.* **11**, 23963 (2021). 34907208 10.1038/s41598-021-03269-w PMC8671402

[22] Bracher, J., Ray, E. L., Gneiting, T. & Reich, N. G. Evaluating epidemic forecasts in an interval format. *PLoS Comput. Biol.* **17**, e1008618 (2021). 33577550 10.1371/journal.pcbi.1008618 PMC7880475

[23] Taylor, J. W. & Taylor, K. S. Combining probabilistic forecasts of Covid-19 mortality in the United States. *Eur. J. Oper. Res.* **304**, 25–41 (2023). 34219901 10.1016/j.ejor.2021.06.044 PMC8236414

[24] Ray, E. L. et al. Comparing trained and untrained probabilistic ensemble forecasts of COVID-19 cases and deaths in the United States. *Int. J. Forecast.* **39**, 1366–1383 (2023). 35791416 10.1016/j.ijforecast.2022.06.005 PMC9247236

[25] Houtekamer, P. The quality of skill forecasts for a low-order spectral model. *Mon. Weather Rev.* **120**, 2993–3002 (1992).

[26] Rahmandad, H., Xu, R. & Ghaffarzadegan, N. Enhancing long-term forecasting: Learning from COVID-19 models. *PLoS Comput. Biol.* **18**, e1010100 (2022). 35587466 10.1371/journal.pcbi.1010100 PMC9119494

[27] Whitaker, J. S. & Loughe, A. F. The relationship between ensemble spread and ensemble mean skill. *Mon. Weather Rev.* **126**, 3292–3302 (1998).

[28] Shaman, J. & Karspeck, A. Forecasting seasonal outbreaks of influenza. *Proc. Natl. Acad. Sci.* **109**, 20425–20430 (2012). 23184969 10.1073/pnas.1208772109 PMC3528592

[29] Bergström, F., Günther, F., Höhle, M. & Britton, T. Bayesian nowcasting with leading indicators applied to Covid-19 fatalities in Sweden. *PLOS Comput. Biol.* **18**, e1010767 (2022). 36477048 10.1371/journal.pcbi.1010767 PMC9762573

[30] Kitaoka, T. & Takahashi, H. Improved prediction of new COVID-19 cases using a simple vector autoregressive model: Evidence from seven New York State counties. *Biol. Methods Protoc.* **8**, bpac035 (2023). 36741926 10.1093/biomethods/bpac035 PMC9893212

[31] Alabdulrazzaq, H. et al. On the accuracy of ARIMA-based prediction of COVID-19 spread. *Results Phys.* **27**, 104509 (2021). 34307005 10.1016/j.rinp.2021.104509 PMC8279942

[32] Küchenhoff, H., Günther, F., Höhle, M. & Bender, A. Analysis of the early COVID-19 epidemic curve in Germany by regression models with change points. *Epidemiol. Infect.* **149**, e68 (2021). 33691815 10.1017/S0950268821000558 PMC7985895

[33] Spreco, A. et al. Nowcasting (short-term forecasting) of COVID-19 hospitalizations using syndromic healthcare data, Sweden, 2020. *Emerg. Infect. Dis.* **28**, 564 (2022). 35201737 10.3201/eid2803.210267 PMC8888224

[34] Gudde, A. et al. Predicting hospital admissions due to COVID-19 in Denmark using wastewater-based surveillance. *Sci. Total Environ.* **966**, 178674 (2025). 39904216 10.1016/j.scitotenv.2025.178674

[35] Abbott, S. et al. Epinow2: Estimate real-time case counts and time-varying epidemiological parameters. https://zenodo.org/records/14899316 (2025).

[36] Raftery, A. E., Gneiting, T., Balabdaoui, F. & Polakowski, M. Using Bayesian model averaging to calibrate forecast ensembles. *Mon. Weather Rev.* **133**, 1155–1174 (2005).

[37] Howerton, E. et al. Context-dependent representation of within-and between-model uncertainty: Aggregating probabilistic predictions in infectious disease epidemiology. *J. R. Soc. Interface* **20**, 20220659 (2023). 36695018 10.1098/rsif.2022.0659 PMC9874266

