# Supplementary material for: Evaluation of respiratory disease hospitalisation forecasts using synthetic outbreak data
Source: Commun Med (Lond). 2026 Jul 29;6:420. doi: 10.1038/s43856-026-01802-4 (PMC13421584; doi:10.1038/s43856-026-01802-4)
Supplement: Supplementary file 2 — Supplemental Information [file 43856_2026_1802_MOESM2_ESM.pdf]

Supplementary Information for "Evaluation of respiratory disease hospitalisation forecasts using synthetic outbreak data"

Grégoire Béchade[1,*], Torbjörn Lundh[2], and Philip Gerlee[3]

[1]Ecolé Polytechnique, gregoire.bechade@polytechnique.edu

[2]Department of Mathematical Sciences, Chalmers University of Technology & University of Gothenburg, Sweden, torbjorn.lundh@chalmers.se

[3]Department of Mathematical Sciences, Chalmers University of Technology & University of Gothenburg, Sweden gerlee@chalmers.se

[*]Corresponding author: Philip Gerlee

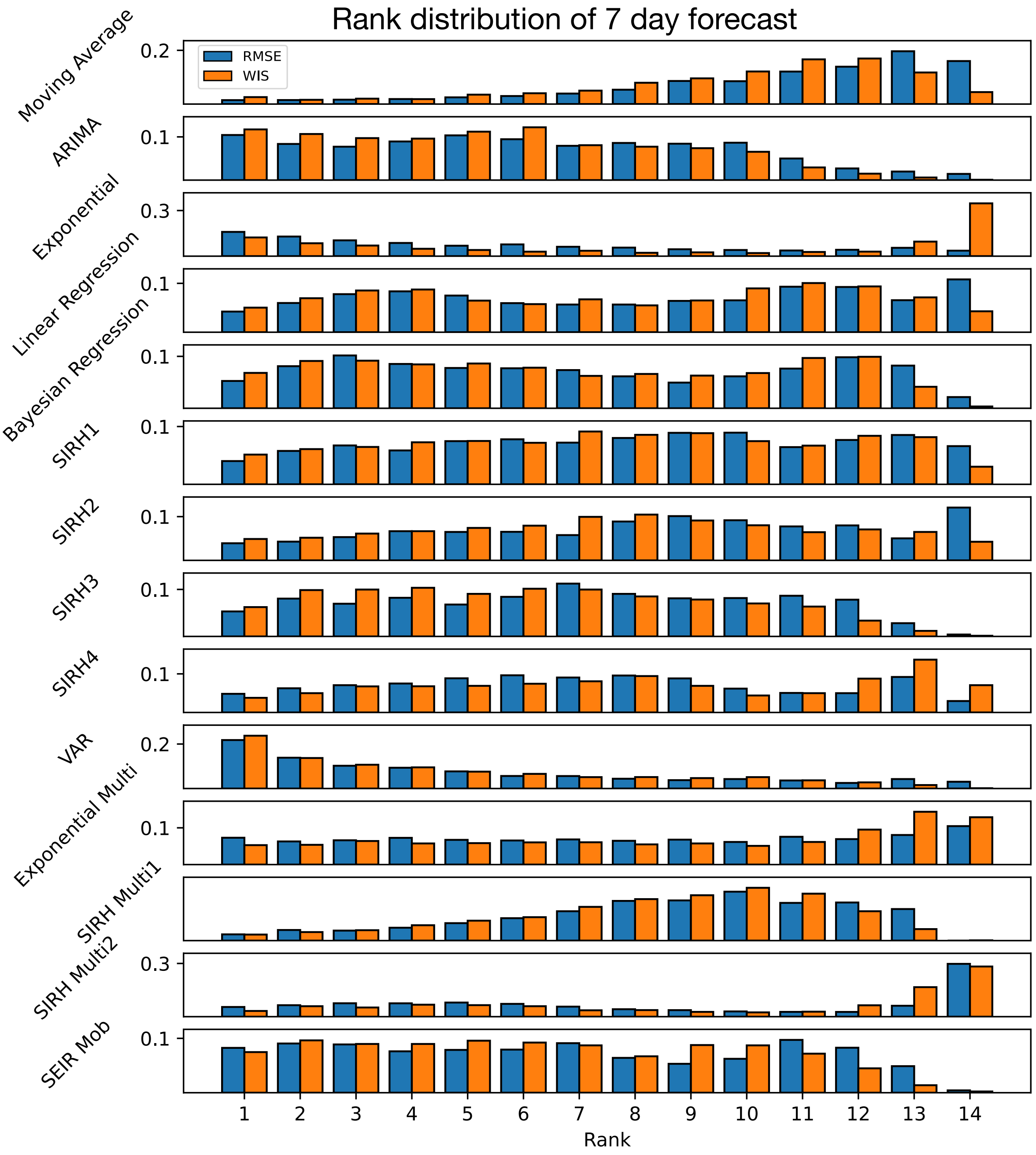

Supplementary Figure 1: Distribution of rankings of the models for all points for 7-day forecasts with respect to both RMSE and WIS.

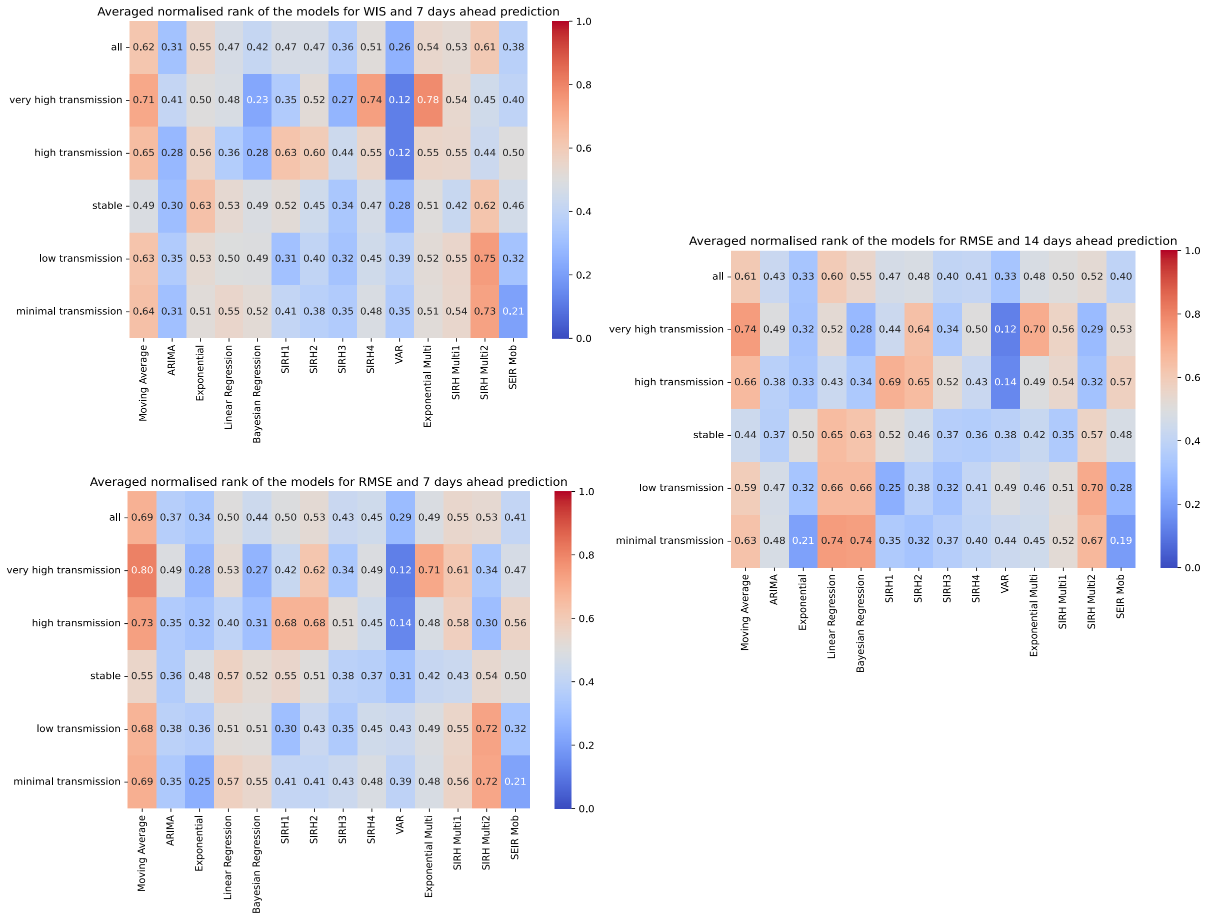

Supplementary Figure 2: Heatmap of model performance based on RMSE and WIS for 7- and 14-day forecasts. The day of forecast is classified according to the current effective reproduction number according to: minimal transmission ($R_{\text{eff}} < 0.5$), low transmission ($0.5 \leq R_{\text{eff}} < 0.8$), stable ($0.8 \leq R_{\text{eff}} < 1.2$), high transmission ($1.2 \leq R_{\text{eff}} < 3$) and very high transmission $R_{\text{eff}} \geq 3$.

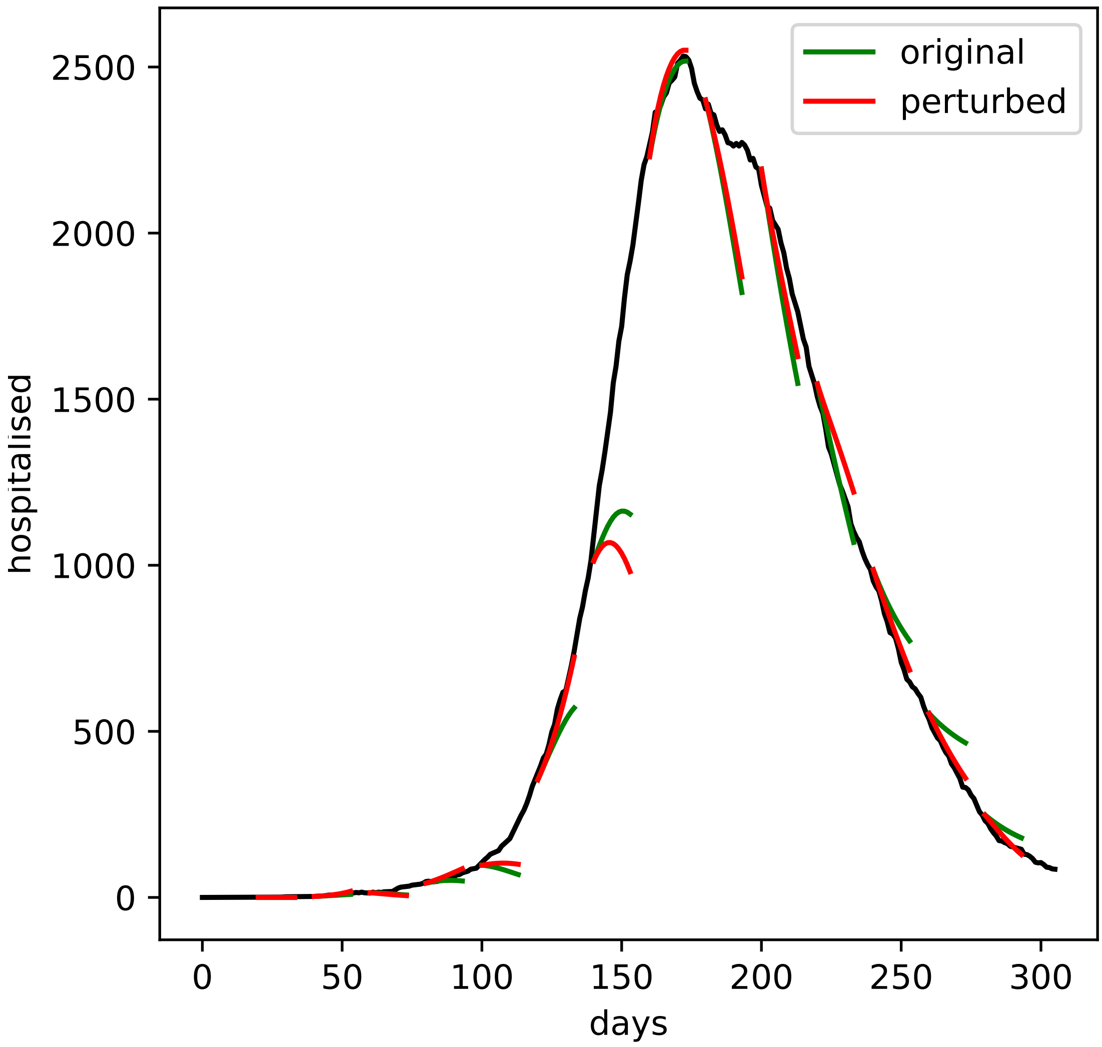

Supplementary Figure 3: Example outbreak where the SEIR Mob-model which uses mobility data updated every 20 days (red curves) performs better than the original SEIR Mob-model which uses mobility updated daily (green).

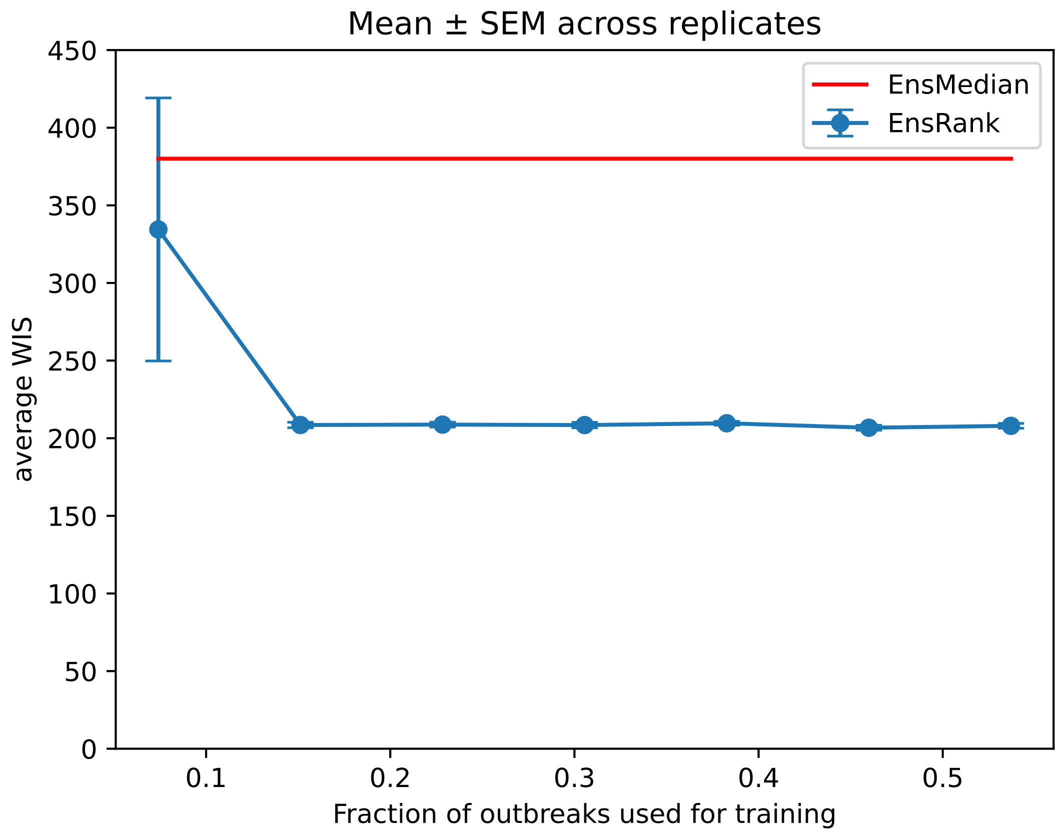

Supplementary Figure 4: The performance of EnsRank in terms of average WIS for 14-day forecasts as a function of the fraction of outbreaks used for calculating the ensembles weights. Circles show the average over 10 realisations and errors bars correspond to standard error of the mean. For reference the red line shows the average WIS of EnsMedian which is independent of the fraction of outbreaks.

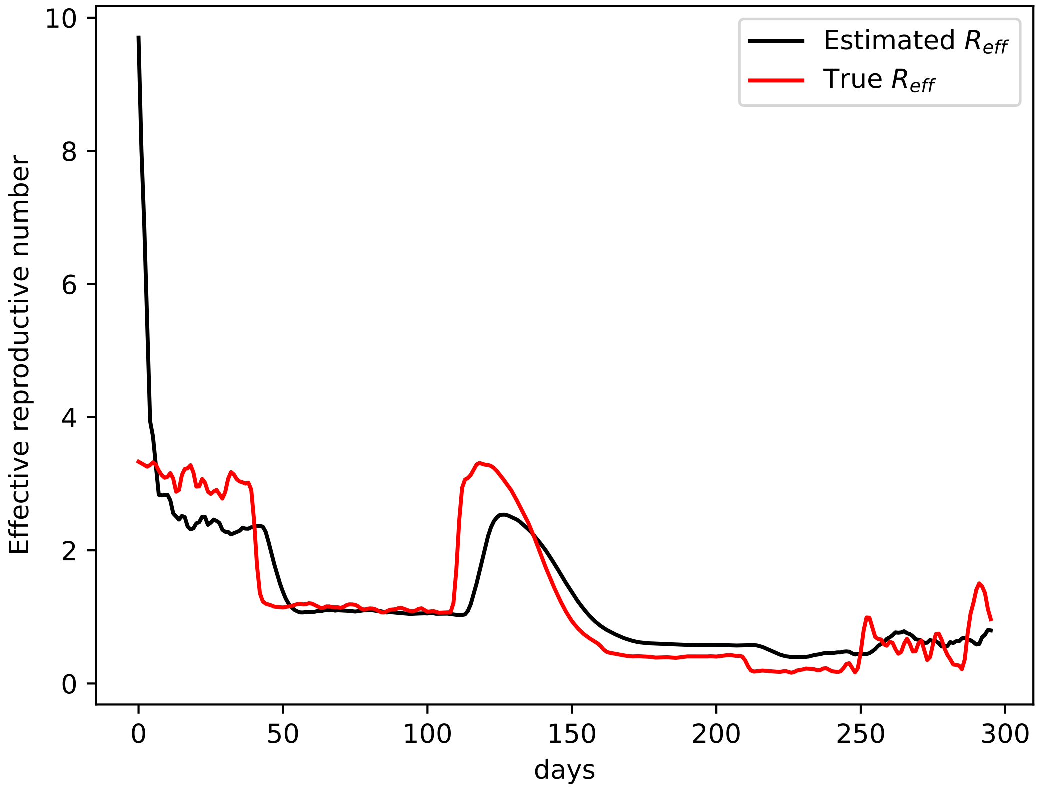

Supplementary Figure 5: An example of estimated and true effective reproductive number for a single Covasim outbreak. See Methods for details concerning the estimation.

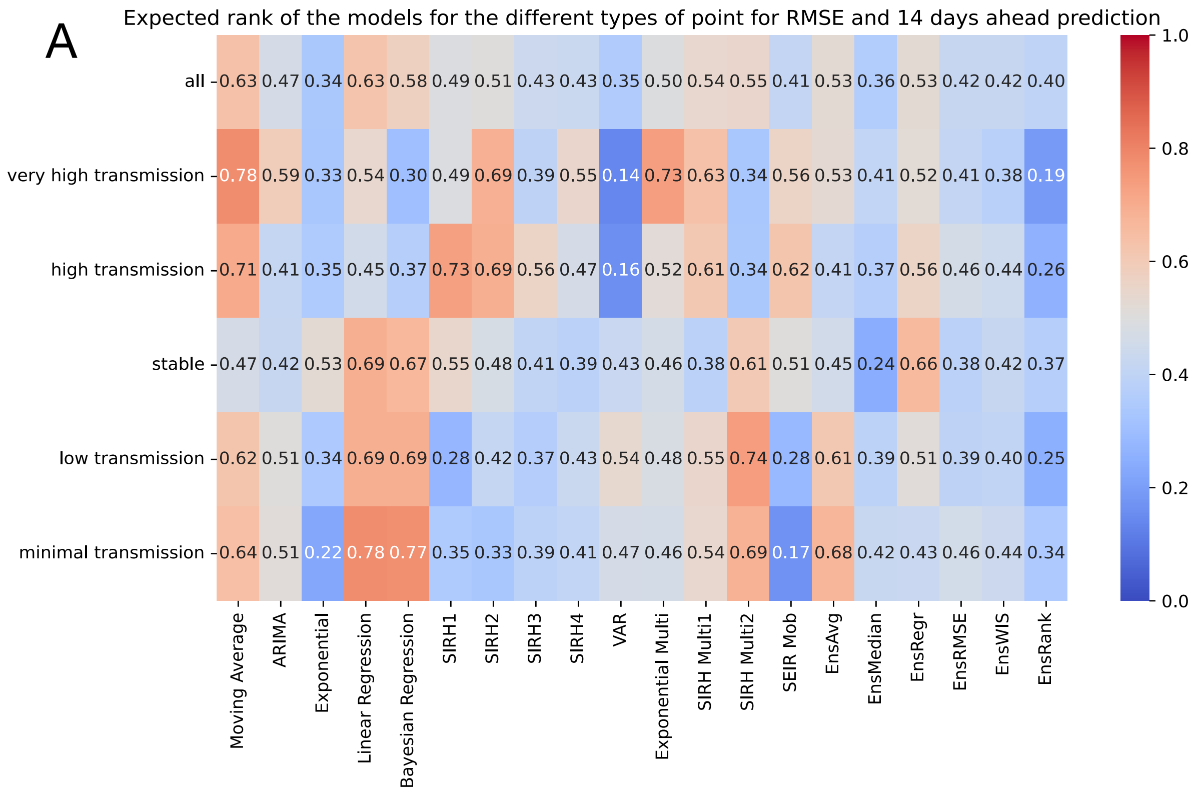

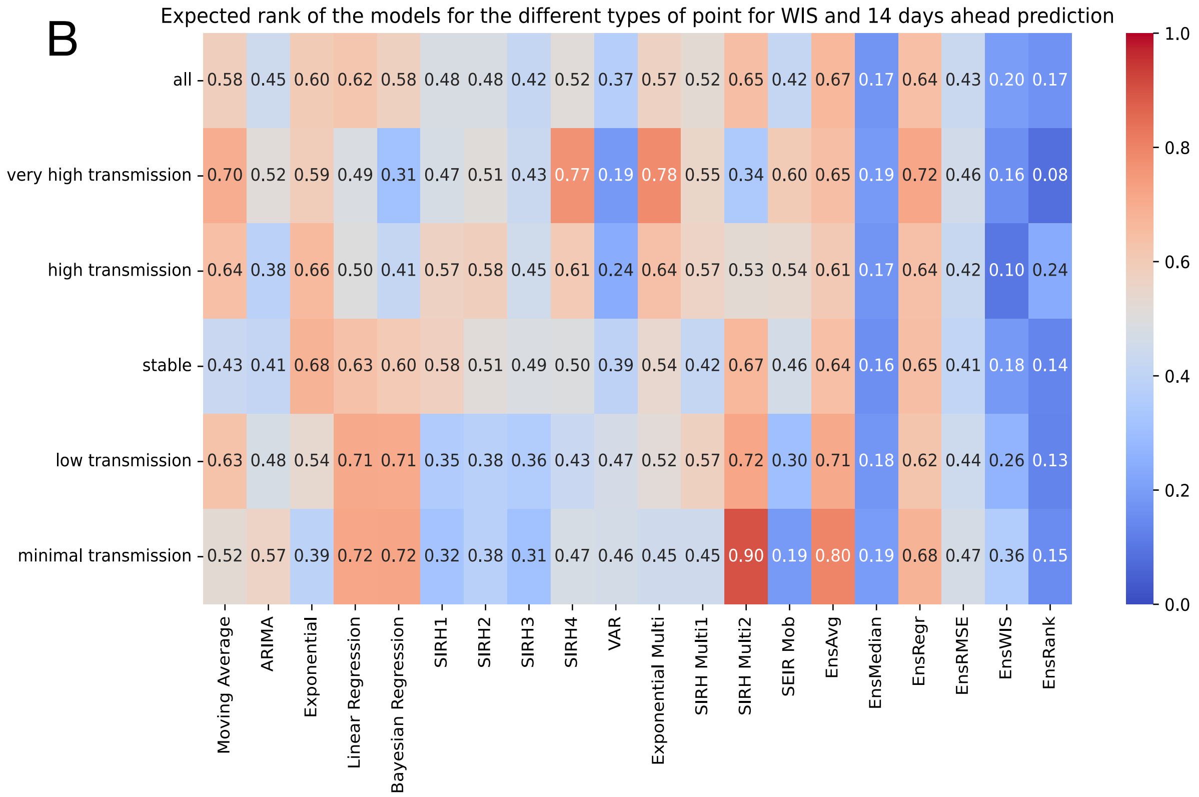

Supplementary Figure 6: Heatmap of model performance based on RMSE and WIS for 7- and 14-day forecasts when estimated R_{eff} is used instead of the true R_{eff} taken from Covasim. The day of forecast is classified according to the current effective reproduction number according to: minimal transmission ($R_{\text{eff}} < 0.5$), low transmission ($0.5 \leq R_{\text{eff}} < 0.8$), stable ($0.8 \leq R_{\text{eff}} < 1.2$), high transmission ($1.2 \leq R_{\text{eff}} < 3$) and very high transmission $R_{\text{eff}} \geq 3$.

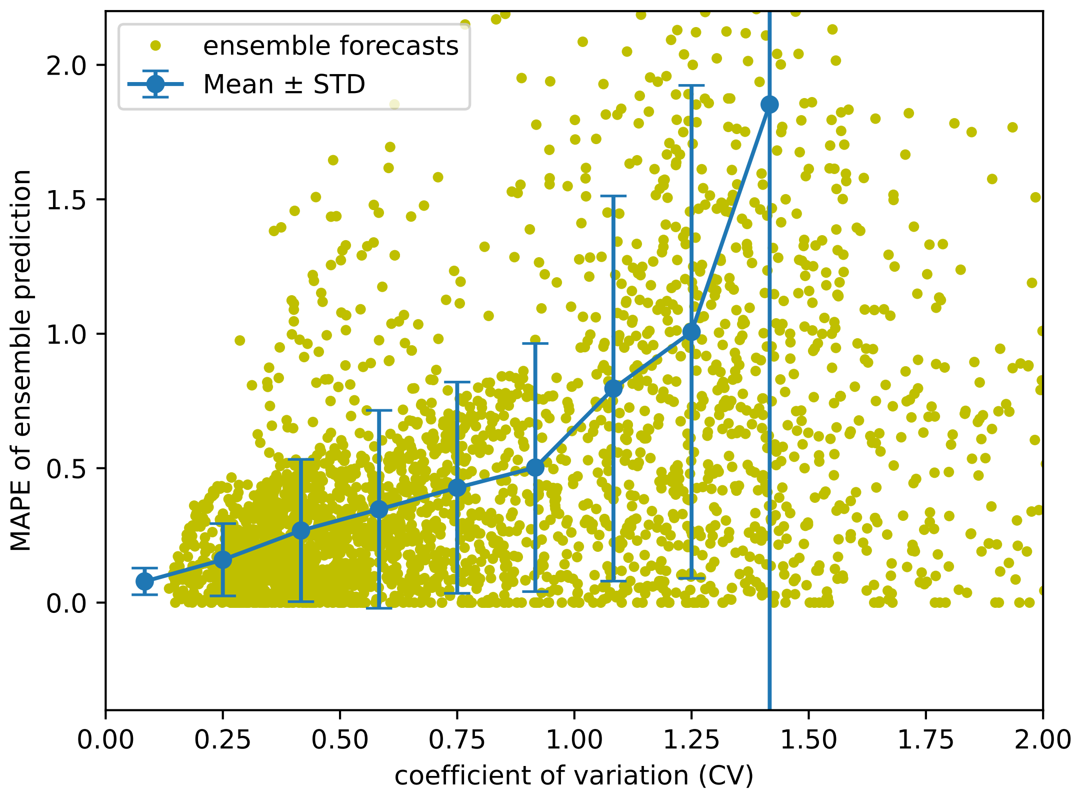

Supplementary Figure 7: The coefficient of variation (CV) of the individual model predictions for 14-day forecasts and the corresponding error (MAPE) of the Median Ensemble prediction. The solid line shows the mean MAPE in each bin and the error bars correspond to one standard deviation.

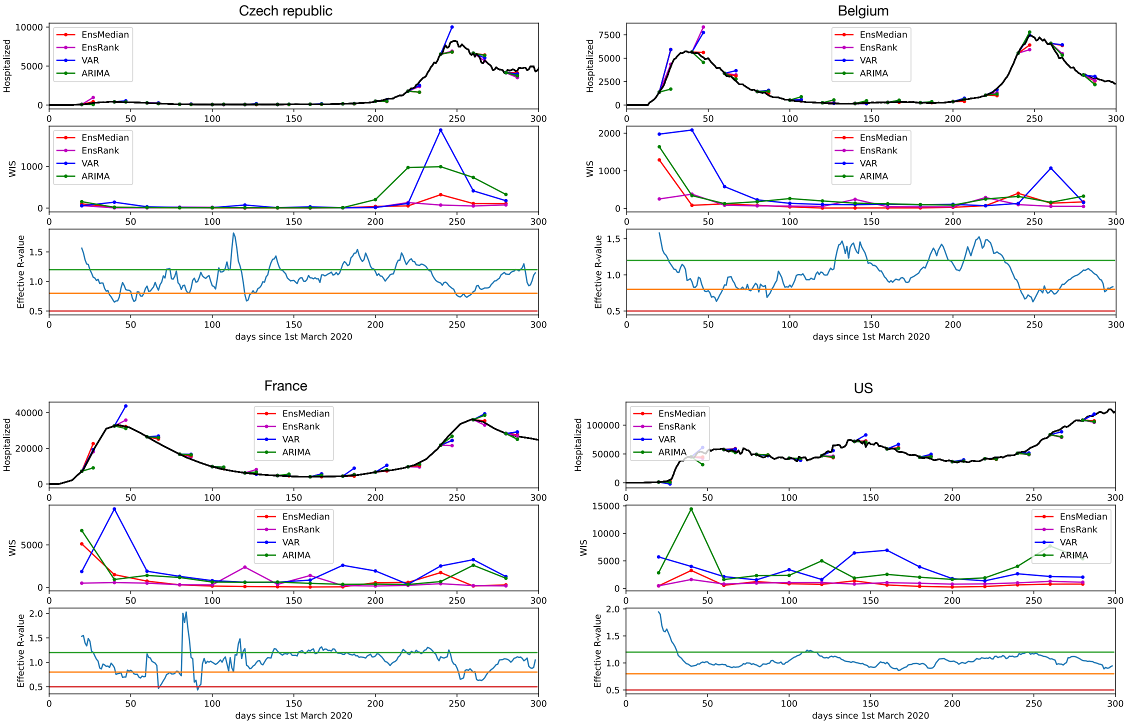

Supplementary Figure 8: Evaluation of selected component model and ensemble 7-day forecasts for COVID-19 data from Czech republic, Belgium, France and the US during 2020. A) Point predictions from the best performing ensembles (median- and adaptive rank-based) and component models (VAR and ARIMA). B) The weighted interval score (WIS) of the ensembles and models calculated from prediction intervals for each forecast. C) Estimate of effective reproduction value. The solid lines correspond to break points for the adaptive rank-based ensemble. The red line corresponds to $R_{\text{eff}} = 0.5$, the orange line to $R_{\text{eff}} = 0.8$ and the green line to $R_{\text{eff}} = 1.2$

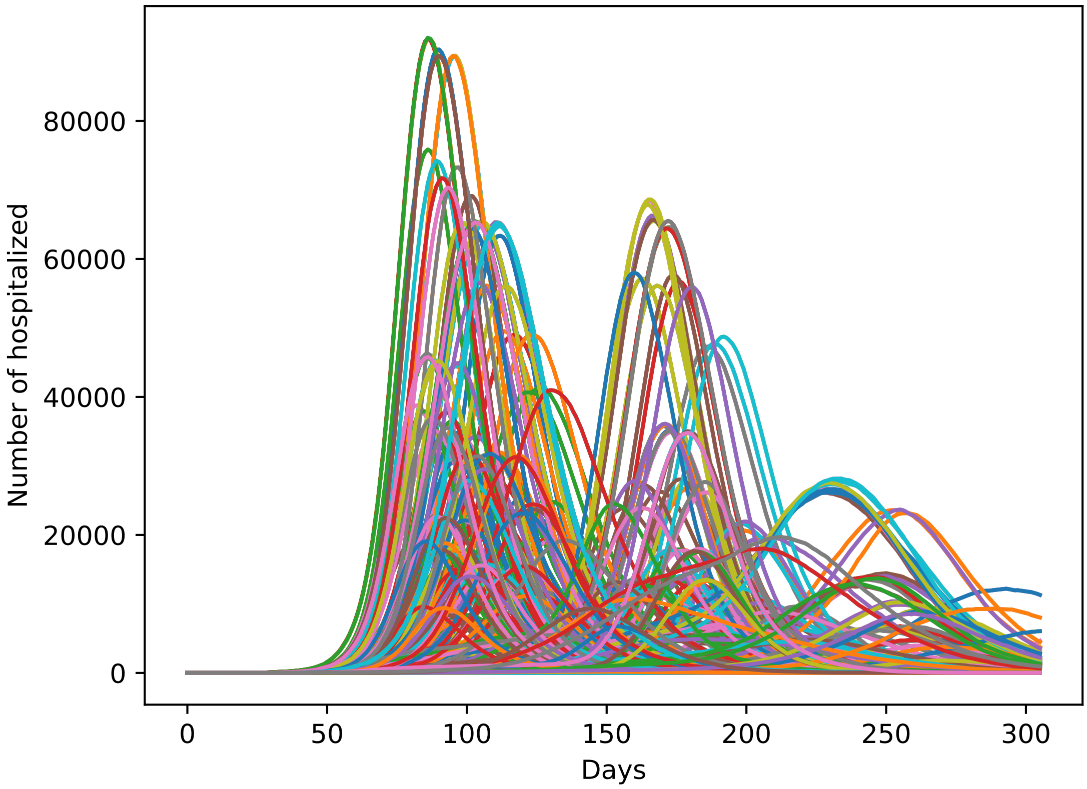

Supplementary Figure 9: All the hospitalisation curves generated from Covasim used for evaluation.

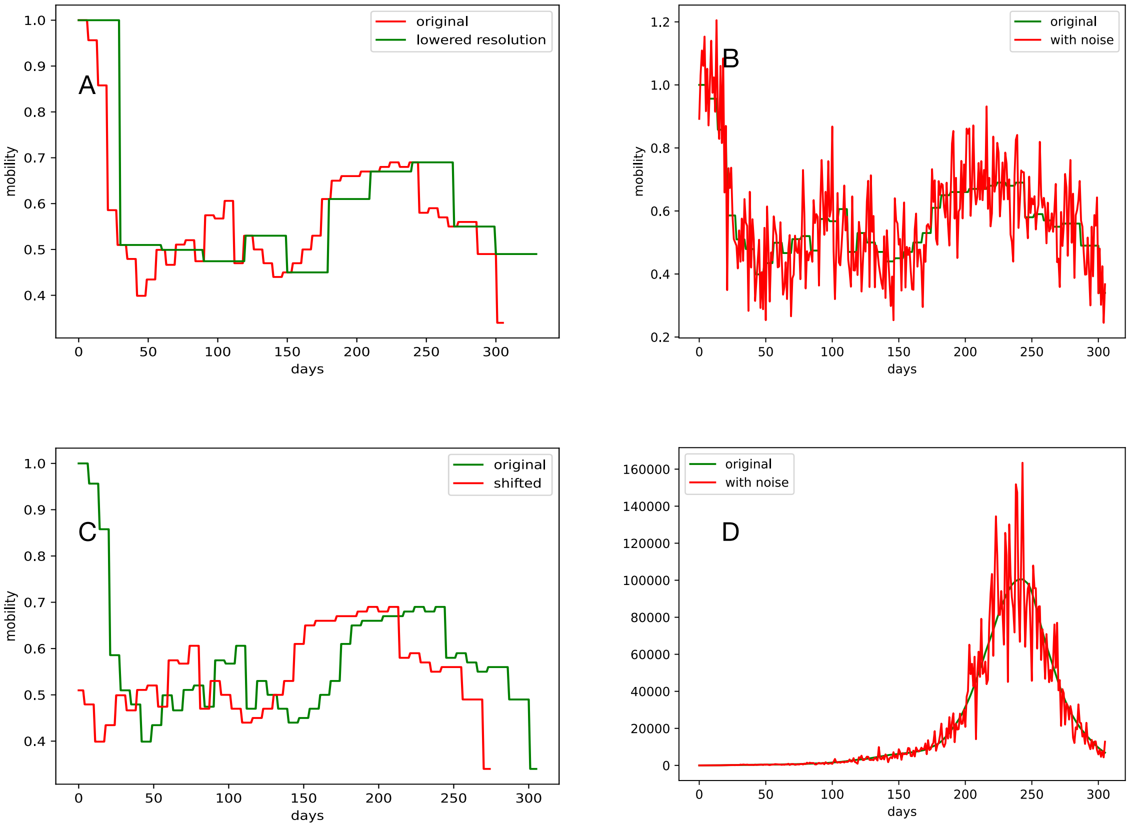

Supplementary Figure 10: The different types of perturbations applied to the additional data streams shown at their extremes. A The temporal resolution of the data is reduced and only updated every 30 days. B Uncorrelated noise is added to the mobility. C The data is delayed by 30 days. D Uncorrelated noise is added to the incidence data.
