# Supplementary material for: Evaluation of respiratory disease hospitalisation forecasts using synthetic outbreak data
Source: Commun Med (Lond). 2026 Jul 29;6:420. doi: 10.1038/s43856-026-01802-4 (PMC13421584; doi:10.1038/s43856-026-01802-4)
Supplement: Supplementary file 1 — Transparent Peer Review file [file 43856_2026_1802_MOESM1_ESM.pdf]

nature portfolio

Peer Review File

Evaluation of respiratory disease hospitalisation forecasts using synthetic outbreak data

Corresponding Author: Professor Philip Gerlee

This manuscript has been previously reviewed at another journal. This document only contains information relating to versions considered at Communications Medicine.

This file contains all reviewer reports in order by version, followed by all author rebuttals in order by version.

Version 1:

Reviewer comments:

Reviewer #1

(Remarks to the Author)
I would expect a point-by-point response rather than a summary. This revised manuscript represents a clear improvement. The authors present a comprehensive and carefully designed evaluation of short-term hospitalisation forecasts using both synthetic outbreak data and real-world COVID-19 data from several countries. Many of the concerns raised in the first review round have been addressed convincingly. In particular, the description of the training–evaluation split and the expanding-window design is now clearer; the set of performance metrics has been expanded to include MAE and MAPE; and the real-data evaluation has been strengthened and better documented. The Discussion has also been improved and now engages more explicitly with issues of robustness, data quality, and the limits of what synthetic experiments can tell us about real-world deployment. The remaining comments I provide below are therefore relatively minor and mostly concern clarification.

1. The authors state that in the multivariate exponential regression model "we assume that the mobility and incidence take last known value for all future dates" (around Line 600–604). The authors also describe linear extrapolation of mobility beyond the forecast origin for mechanistic models using mobility, e.g. SIRH-Multi "we make use of a linear extrapolation of the mobility since its future values are unknown to the forecaster" (around Line 685–688), and in the Discussion you again refer to linear extrapolation for models that utilise additional data streams. (around Line 459-463)
It would be helpful to clearly distinguish which models use "last observed value carried forward" versus linear extrapolation, and to ensure this is described consistently across Methods and Discussion.

2. In the simulation study, one of the four mobility patterns used in CovaSim is the empirical Google mobility time series from Sweden, alongside more stylised patterns (seasonal, lockdown, constant). In Section 4.10 the real-world evaluation then uses hospitalisation and mobility data from Sweden (and other countries).
This means that the real-data performance for Sweden is, in part, being assessed in a setting where the synthetic training set already contains a mobility pattern very similar to that real series. While I appreciate that you now explore robustness to noise, delays and temporal resolution in the additional data streams, this structural overlap could still be mentioned explicitly as a limitation.

3. Section 4.10 now provides data sources for hospitalisations and mobility for Sweden, and some other countries. Adding 1–2 sentences on implementation details would strengthen reproducibility of the real-data evaluation and make it easier to compare performance across countries.

4. In Section 4.4 the authors introduce three performance metrics (WIS, RMSE and MAPE) as primary evaluation criteria (Lines 702–724). However, several key summaries and discussions still focus exclusively on RMSE and WIS. Given that a relative error measure was specifically requested and MAPE is now computed for all models, it would be helpful either to integrate MAPE more directly into the main comparative results, or to briefly justify why the narrative focuses primarily on RMSE and WIS.

5. (Line 629-629) the last sentence is duplicated: "The prediction intervals are also directy provided by the library. The prediction intervals are also directy provided by the library." It has "directy" instead of "directly".

6. Also, some changes are still unclear. For example, the author say the use estimated Rt instead of the true Rt, did they mimic the real situation that they can only use the data up to time t to estimate Rt? if so, how they account for the censoring issue and fluctuation on Rt for the most recent day? did they use the point estimate? Would CI play a role?

Reviewer #2

(Remarks to the Author)
I co-reviewed this manuscript with one of the reviewers who provided the listed reports. This is part of the Communications Medicine initiative to facilitate training in peer review and to provide appropriate recognition for Early Career Researchers who co-review manuscripts.

Reviewer #3

(Remarks to the Author)
I am satisfied with the changes that the authors have made and am happy to recommend this manuscript for publication.

Version 2:

Reviewer comments:

Reviewer #1

(Remarks to the Author)
Thank you. I have no further comment.

Reviewer #2

(Remarks to the Author)
I co-reviewed this manuscript with one of the reviewers who provided the listed reports. This is part of the Communications Medicine initiative to facilitate training in peer review and to provide appropriate recognition for Early Career Researchers who co-review manuscripts.

Open Access This Peer Review File is licensed under a Creative Commons Attribution 4.0 International License, which permits use, sharing, adaptation, distribution and reproduction in any medium or format, as long as you give appropriate credit to the original author(s) and the source, provide a link to the Creative Commons license, and indicate if changes were made.
In cases where reviewers are anonymous, credit should be given to 'Anonymous Referee' and the source.
The images or other third party material in this Peer Review File are included in the article’s Creative Commons license, unless indicated otherwise in a credit line to the material. If material is not included in the article’s Creative Commons

license and your intended use is not permitted by statutory regulation or exceeds the permitted use, you will need to obtain permission directly from the copyright holder.
To view a copy of this license, visit https://creativecommons.org/licenses/by/4.0/

Reply to reviewers' comments

We would like to thanks all reviewers for the detailed and constructive criticism of the manuscript. The resulting changes have strenghtened the results and improved the overall quality of the manuscript. Below we provide a list of implemented changes that relate to both major and minor comments.

Major changes

1. Reviewer #1, #2 & #4 raised the concern that testing on real data was limited to a single data set. We have remedied this limitation by testing our methods on additional data sets, including COVID-19 hospitalisations from France, Belgium, Czech Republic and the United States. This additional analysis, which includes all component models and ensembles, shows that the median ensemble has the lowest average rank for 3 out of 4 for performance targets (see Section 2.4 and Table 3), while the rank-based ensemble performs best for 7-days forecasts in terms of RMSE. However, the rank-based ensemble outperforms the median ensemble in 40% of 14-day forecasts (and 27% of 7-day forecasts). These new results have been added to the Results and are also mentioned in the Discussion.
2. Reviewer #2 was concerned that we used 50% of the Covasim data to train the ensemble weights. We have investigated how the rank-based ensemble performs in terms of average WIS as a function of the fraction of outbreaks used in training. The results show that performance remains constant until approximately 15% of the Covasim data is used, below which performances degrades rapidly (see Supplementary fig. 4 and L269-275).
3. Reviewer #2 suggested that we evaluate all models on the real data sets. This analysis has been carried out and is presented in Table 3 and 4 (see point 1 above).
4. Reviewer #2 & #4 suggested that a relative measure of forecast error (e.g. MAPE) should be used alongside RMSE and WIS. We have implemented this and present trimmed MAPE for both the synthetic data and the real COVID-19 dataset (see Table 2 and 4 and accompanying text).
5. Reviewer #2 raised concern regarding the fact that we use the true effective reproductive number (R_eff) to inform the rank-based ensemble (EnsRank). We have investigated how EnsRank performs when we instead estimate R_eff from incidence data from Covasim and the results are very similar and importantly EnsRank still has

the lowest average normalised rank when WIS is used as an evaluation metric. The results can be seen in supplementary fig. 6.

6. Reviewer #4 suggests that the impact of the additional data streams should be investigated by perturbing them in ways that are more realistic. When carrying out this analysis we found an unfortunate interaction between the interpolation carried out when reducing the temporal resolution of the data streams and the extrapolation used by the mechanistic models. To avoid this effect have now removed the interpolation step when down-sampling the data streams. Despite this the SEIR-Mob model performs slightly better on the down-sampled mobility data (see supp. fig. 3). We hypothesise that this occurs because improvement in training out-weighs the reduction in accuracy due to the mobility data changing less often (see L402-410). We have also amended the analysis by investigating the performance of the models when the data streams are subject to a lag and noise. We have incorporated the results of this analysis into fig. 5, now containing four panels. The details of the perturbations are described in the Methods (L747-759).

Minor changes

Reviewer #1:

On L318 the authors state "A benefit of our approach with a large-scale synthetic, yet realistic, data set is that the results we obtain are more reliable compared to those obtained from a single epidemic". I find this to be an unfounded statement based on the work presented. "Reliable" is not defined and, at best, is tested relative to a single observed outbreak.

The sentence has been rephrased to read: "A benefit of our approach with a large-scale synthetic, yet realistic, data set is that it is possible to observe statistical trends not observable in a single epidemic".

While the authors generated a wide range of synthetic scenarios, all are limited to an emergent outbreak in a completely susceptible population.

The models that we have developed are indeed constructed for an outbreak in a completely susceptible situation. We did evalute the performance on time-series of hospitalisations due

to influenza in the US during the 2021/2022 season (selected due to the availability of Google Mobility Reports). However, we noted that the mechanistic models performed poorly in this setting, while the statistical models exhibited acceptable performance. For a fair comparison we would need to consider a different set of mechanistic models that are suitable for seasonal influenza. In order to not detract from the main message of the manuscript we have decided not go down this route and instead reserve this for future work. We now mention this possibility in the Discussion (L477-482) and also clarify that our focus is emergent outbreak in the Introduction (L89).

Reviewer #2

The synthetic data were generated using parameters similar to COVID-19 dynamics. These parameters were only known retrospectively. This limits the method's utility in early-stage novel outbreaks, or outbreaks with change of epidemiology due to new variants, where such parameters are unknown or highly uncertain.

We now clarify that partial knowledge about the parameters from Covasim is only used by a subset of the mechanistic forecasting models and never used (retrospectively) during evaluation. We only make use of the hospitalisation curves produced by Covasim. (L133-136).

Real Swedish mobility data was used to generate synthetic outbreaks, and Swedish hospitalization data was later used for validation. While real hospitalization outcomes weren't used in training, this may introduce structural bias favoring models tuned to Sweden-like dynamics. Furthermore, if those mobility data cannot available in real-time, there can be potential look-ahead bias.

While in theory the use of the same mobility data both for training and validation could introduce a certain bias we deem this unlikely since the mobility has an indirect effect on hospitalisations. We now quantify the effects of noise, delay and temporal down-sampling of the mobility data (see fig. 5). The observation is that the mechanistic model SEIR-Mob model is less sensitive to these perturbations compared to the auto-regressive VAR-model.

The R_eff stratification thresholds (0.5, 0.8, 1.2, 3.0) appear to be arbitrarily chosen without theoretical justification or literature support. While R_eff = 1.0 as the epidemic growth threshold is well-established, the other cutpoints lack clear rationale.

We agree that the location and also the number thresholds are arbitrary chosen. However, investigating how this choice affects the performance of the rank-based ensemble is a considerable effort in itself and will be the focus of future work. We now mention this in the discussion. (L488-492)

The assumption that mobility and incidence remain constant at their last known values for future predictions is unrealistic, as these variables typically exhibit significant variation during epidemics due to policy interventions and behavioral changes. This limitation should be discussed, and its impact on multivariate model performance should be acknowledged.

We were not clear about the fact that the forecast models make use of a linear extrapolation of mobility and incidence. This has been clarified and we now discuss this limitation and future extensions in the Discussion (L459-464).

While all models use data through day t, the mechanistic models can benefit from an additional "shifting" step that forces H(t)= Y_t. This differs from how statistical models naturally incorporate day t information.

We acknowledge this difference which is readily explained in the Methods section (L681-683).

The Average Ensemble (EnsAvg) is constructed as an unweighted mean over all 14 component models, regardless of their individual accuracy. This is rarely done in practice, as poor-performing models can disproportionately degrade ensemble performance.

Our intention is to present a suite of ensemble methods that range from the simplest (EnsAvg) to more sophisticated methods (EnsRank) and compare their performance. Despite its overly simple construction EnsAvg still outperforms 1/3 of all models on average, which we think merits its inclusion. In addition, the unweighted mean was used by the US COVID-19 Forecast Hub during the early stages of the pandemic.

Most ensemble methods use fixed weights throughout entire epidemic curves (300 days), ignoring potential changes in model performance as epidemic conditions evolve.

It would be interesting to see how an adaptive ensemble would compare to the ensemble methods we consider. We now mention this extension in the Discussion. (L488-492)

There is an inconsistency in the text. It would be helpful to clarify whether forecasts are made "every 20 days" or "every 10 days".

The sentence has been reformulated and we have made clear that we make forecasts every 20 days.

Supplementary Figure 3 should be moved to the main paper instead of Figure 7, as it provides a more thorough and direct comparison between all models including both individuals and ensembles.

We agree that the figure in the supplement is more informative and it has been moved to the main text.

It would be helpful to clarify the two different numbers of evaluation points (4860 and 2549). (Line 272-275,602-604)

The number reported in the main text was erroneous and has now been corrected.

Reviewer #4

For example, I was interested in how the model weights in Fig 4A might be different to optimize the forecast performance of the different ensembles (other than EnsMedian and EnsRank). E.g., for EnsReg do the same coefficients make the best form of that type of ensemble. . It seems like results like this would be important for establishing whether the results based on the synthetic data extend to real epidemics. Further description and discussion of how EnsRank is developed in the applied data setting would also be helpful.

It is not quite clear what the reviewer is referring to (there is no fig. 4A with ensemble weights). The point we are trying to make is that during an ongoing outbreak there is not enough data to optimise an ensemble with respect to component model weights. The alternative we propose is to use synthetic data for ensemble optimisation, which can then be applied at the start of a novel outbreak. We now discuss the potential and limitations of this approach in the Discussion (L382-393).

However, I think there is a bit of a stretch in lines 300-302 where it is not expressed how difficult this approach would be in practice and it doesn't seem that the connection between improved performance for the simulated dataset and improved performance for real world datasets has been fully established.

The paragraph in question has been rewritten and gives a more balanced account of the benefits of the approach.

Some aspects of the methods could be better explained or expanded to increase reproducibility. Line 531 of the methods could be expanded to increase reproducibility. It's also not clear why 1/8 and 1/18 are chosen as gamma values in SIRH-Multi methods starting on line 552.

The section on Bayesian regression has been rewritten for clarity and we now explain reasoning behind the default values in the SIRH-models.

Line 34: relevant timescales also dependent on the goals of the exercise, e.g., is the impact of an intervention going to be analyzed. Could reference the designing scenarios paper here for projections.

We now cite the suggested paper by Runge et al.

Line 61: Recommend not naming the US COVID-19 Forecast Hub as the most prominent initiative, since this is subjective. Suggest also referencing the following which documents recent FluSight Influenza Forecast Hub results and perhaps acknowledging that this effort has been running since the 2013/2014 influenza season.
Mathis, Sarabeth M., et al. "Evaluation of FluSight influenza forecasting in the 2021–22 and 2022–23 seasons with a new target laboratory-confirmed influenza hospitalizations." Nature communications 15.1 (2024): 6289.

We now mention the US COVID-19 Forecast as an example among others and also mention and cite the FluSight Challenge.

Line 115: It would be helpful to comment on whether the produced parameter ranges that would halved or doubled were biologically plausible and perhaps mention that the magnitude of change for one parameter may have a larger effect than the same change for another parameter.

We now comment on the plausibility of altering the parameter values (L560-563).
Concerning the relative impact of changing the parameters we have solved this problem by defining the outbreak metric (eq. 1) which makes it possible to identify parameter sets that

yield large changes to the outbreak when changed alone or in combination. To highlight the diversity of outbreaks we now include panel 1B as a stand alone supplementary figure 9.

Line 118: Better to state that you defined epidemics to be 300 days long rather than yielding epidemics of that length. Or say how you are determining the epidemic length. It may also be helpful to state here or elsewhere before currently stated that results are truncated to not include days with less than 10 counts.

The epidemics might be longer than 300 days, in order to make the forecasting comparable across all outbreaks we terminate the hospitalisation curves after 300 days. This is now explained in the manuscript. (L566-570)

Line 124-125: Suggest expanding in the discussion on how feasible these types of models would be for real-time real-world applications.

We now discuss this at L382-393 in the Discussion.

Line 133: It would be better to quantify what constitutes as a "good forecast".

We have reworded to "accurate forecast".

Figure 2: It's unclear what (blue at day 0 and red at day 14) means. Should these be points instead of lines? Are they just the forecasts at specific time points? The lines give the impression that you are continuously forecasting over that full interval.

We now state in the figure text that "The colouring of the forecast curves is meant to increase readability and indicates the length of the forecast (blue at day 0 and red at day 14)".

Line 141: Can you say anything more about the "small fraction of cases" where MA does perform best? Do they have a unifying feature?

We have not looked into detail concerning this, but since MA makes a flat forecast based on the preceeding 7 days it most likely performs best when hospitalisations remain constant. We plan to look into this and the more general question of when specific forecasts fail and

succeed and cluster models based on this. This lies beyond the scope of this manuscript but could improve ensemble forecasting in the future.

Line 153-154: Consider commenting in the discussion why it might be the case that VAR, ARIMA, and SIRH3 perform well with respect to WIS for both the 7 and 14 day forecast.

Unfortunately we don’t know the reason for this. Although it would be interesting to know why, digging into deeper questions like this one is beyond the scope of the manuscript.

Line 174-175: Suggest being careful about making this generalization about the current status of an epidemic being able to inform model choice and how strong the claim is made. Especially with the patchwork of colors in Figure 4 - there are perhaps some generalizations that can be made but there is also some randomness concerning which model performs best in different transmission settings. Also you would need to know the transmission setting ahead of time, in order to operationalize model choice when constructing ensemble models which will likely be difficult in real epidemics.

We now make reference to the paragraph in the Discussion mentioning potential caveats when implementing this in a real-world setting. Concerning the difficulty of operationalising this knowledge we do show that real-time estimation of R-effective using incidence data at the time of forecast can inform the rank-based ensemble, which outperforms all other forecasts. The results from the evaluation on the real-world data, on which the rank-based ensembles performs well, strengthens this observation. However, these results would need to be corroborated in future studies, preferrably in a prospective setting.

Figure 4: It could be helpful to label which models are regression models vs mechanistic or another type along the bottom. It would then be interesting to comment on unexpected rank observations e.g., generally regression models are showing higher rank values (red tones), but the ARIMA model is blue for all levels of transmission. What would cause this difference? What could it be about the VAR model that is leading to the best rank performance for high and very high transmission settings?

These are interesting observations, but slightly outside the scope of the manuscript, and given additional analysis carried out (e.g. adding multiple real-world datasets for evaluation and extending the data perturbation analysis) we decided not to pursue the above questions.

Line 179-180: The unfair comparison between univariate and multivariate models in this case seems like more of a discussion point than a result.

The two sentences are meant as a segue into the question of multiple data streams and temporal resolution.

***Section 2.3 Ensemble model: It may be helpful to consider and reference Howerton, Emily, et al. "Context-dependent representation of within-and between-model uncertainty: aggregating probabilistic predictions in infectious disease epidemiology." Journal of the Royal Society Interface 20.198 (2023): 20220659. and consider a linear opinion pool ensemble, which has become popular recently. A function for calculating the LOP ensemble can be found at* https://hubverse-org.github.io/hubEnsembles/**

We now cite the paper by Howerton et al. in the paragraph on future work in the Discussion.

Line 201-202: Suggest additionally referencing Mathis, Sarabeth M., et al. "Evaluation of FluSight influenza forecasting in the 2021–22 and 2022–23 seasons with a new target laboratory-confirmed influenza hospitalizations." Nature communications 15.1 (2024): 6289.

The reference has been added.

Line 205 and other references to "confidence intervals" throughout: would it be more accurate to write in terms of quantiles and prediction intervals?

We agree that "prediction interval" is more accurate and have changed the notation where appropriate.

Line 239-240: This only seems true if the component models are performing well. If they agree on a value that is different from what is observed the ensemble would also not agree with what is observed.

At this point in the manuscript, we posit a hypothesis that we investigate by comparing the coefficient of variation (CV) and error in terms of MAPE. The analysis shows that on average there is a trend for high CVs of the ensemble to correspond to high MAPE. This observation confirms the above-mentioned hypothesis.

Line 243: Given the first sentence on line 239, I expected that this would be the coefficient of variation across models. Throughout the manuscript it should me made more clear what the coefficient of variation is being calculated over and how this does or doesn't relate to the component models versus the ensembles. On line 249-250 it sounds like the CV is just being calculated over the ensemble values which seems inconsistent with the point of lines 239-240.

We now clarify how the CV is calculated on L288-294.

Line 246/Figure 8: The scatter plot of the MAPE and CV of each individual forecast for all epidemics could be better explained. It isn't clear what is being averaged over and what a single point refers to in figure 8. The description of the coefficient of variation should be consistent between the main text and Figure 8.

See answer above.

Line 318-319: The statement that this approach yields more "reliable" results seems to be an overstatement. Please clarify or reword.

The sentence has been rephrased to read: "A benefit of our approach with a large-scale synthetic, yet realistic, data set is that it is possible to observe statistical trends not observable in a single epidemic".

Line 327-330: It seems like these results about the CV ranges should be included in the results rather than the discussion.

The sentence have been moved to the results.

Instead of the linear interpolation, would a more realistic / comparable version to the real world be to implement a step function interpolation where the same level is used up to 30 days ahead?

We have tried a constant extrapolation, which performs worse than linear extrapolation which is currently used by the mechanistic models that utilise mobility data. We therefore opt for the linear extrapolation when performing forecast. However, we have expanded our analysis concerning the additional data streams (see Major changes point 6). The VAR-

model on the other hand does not use extrapolation, but predicts all three variables (hospitalisations, incidence and mobility) one day ahead and uses these predicted variables to predict the next day and so on. But again the model does not make use of future data points. We now clarify this in the Methods (L625-628 and L687-689).

Reply to comments by Reviewer #1

We would like to thank the reviewer for the detailed and constructive criticism of the manuscript. The resulting changes have strenghtened the results and improved the overall quality of the manuscript. Below we provide a list of implemented changes.

1. The authors state that in the multivariate exponential regression model "we assume that the mobility and incidence take last known value for all future dates" (around Line 600–604). The authors also describe linear extrapolation of mobility beyond the forecast origin for mechanistic models using mobility, e.g. SIRH-Multi "we make use of a linear extrapolation of the mobility since its future values are unknown to the forecaster" (around Line 685–688), and in the Discussion you again refer to linear extrapolation for models that utilise additional data streams. (around Line 459-463)
It would be helpful to clearly distinguish which models use "last observed value carried forward" versus linear extrapolation, and to ensure this is described consistently across Methods and Discussion.

We agree that it was unclear which models used a constant value and which made use of a linear extrapolation. We now clarify this in the Discussion on line 448-451.

2. In the simulation study, one of the four mobility patterns used in CovaSim is the empirical Google mobility time series from Sweden, alongside more stylised patterns (seasonal, lockdown, constant). In Section 4.10 the real-world evaluation then uses hospitalisation and mobility data from Sweden (and other countries).
This means that the real-data performance for Sweden is, in part, being assessed in a setting where the synthetic training set already contains a mobility pattern very similar to that real series. While I appreciate that you now explore robustness to noise, delays and temporal resolution in the additional data streams, this structural overlap could still be mentioned explicitly as a limitation.

Although the mobility data for Sweden was obtained at a regional level from public transport utilisation it most likely has a strong correlation with mobility at the national level and the reviewer rightly points out that this might bias our results. We now clarify the regional origin of the data (line 505-509 and 812-815) and also the limitations it implies (line 453-460).

3. Section 4.10 now provides data sources for hospitalisations and mobility for Sweden, and some other countries. Adding 1–2 sentences on implementation details would strengthen reproducibility of the real-data evaluation and make it easier to compare performance across countries.

We now mentioned that combined CSV-files containing hospitalisations, incidence and mobility for all six countries are available at the Github-repository (line 825-826).

4. In Section 4.4 the authors introduce three performance metrics (WIS, RMSE and MAPE) as primary evaluation criteria (Lines 702–724). However, several key summaries and discussions still focus exclusively on RMSE and WIS. Given that a relative error measure was specifically requested and MAPE is now computed for all models, it would be helpful either to integrate MAPE more directly into the main comparative results, or to briefly justify why the narrative focuses primarily on RMSE and WIS.

We agree that MAPE received little attention in the Discussion and we have now expanded on the performance of the models with respect to MAPE (line 365-370 and 351-353).

5. (Line 629-629) the last sentence is duplicated: "The prediction intervals are also directy provided by the library. The prediction intervals are also directy provided by the library." It has "directy" instead of "directly".

Thank you. This has now been corrected.

6. Also, some changes are still unclear. For example, the author say the use estimated Rt instead of the true Rt, did they mimic the real situation that they can only use the data up to time t to estimate Rt? if so, how they account for the censoring issue and fluctuation on Rt for the most recent day? did they use the point estimate? Would CI play a role?

We now clarify that we estimate the so-called instantaneous reproduction number which only uses data up to the present day to calculate Rt. Since the estimated Rt is used as a threshold value to determine which models to include in the adaptive ensemble we only calculate a point estimate and do not calculate the uncertainty of the estimate. We acknowledge that this approach has certain limitations, e.g. censoring and fluctuating data, and now mention this in the manuscript (line 795-800).
